# Multiblock Analysis of Risk Factors and Management Areas of Calf Mortality in Large-Scale Dairy Herds

**DOI:** 10.3390/ani15192780

**Published:** 2025-09-24

**Authors:** Dagni-Alice Viidu, Triin Rilanto, Stéphanie Bougeard, Tanel Kaart, Kerli Mõtus

**Affiliations:** 1 Institute of Veterinary Medicine and Animal Sciences, Estonian University of Life Sciences, 51006 Tartu, Estonia; 2 Department of Epidemiology, French Agency for Food, Environmental, and Occupational Health Safety, 22440 Ploufragan, France

**Keywords:** dairy calf, on-farm mortality, risk factor, Estonia, multiblock modeling

## Abstract

Despite an abundance of available research, calf mortality persists as a multifaceted phenomenon that presents ongoing challenges in practical management. This historical single-cohort study was conducted to provide a more comprehensive layer of knowledge to the existing information pool on calf mortality risk factors by using multiblock partial least squares analysis. The method reveals the contribution of several variables aggregated into thematic blocks and allows to include multiple outcome variables describing the same phenomenon. Such an analysis of the data provides valuable information to farmers, veterinarians, and advisors alike, not only about single risk factors, but also about management areas to prioritize when tackling calf mortality. Data was gathered from 118 Estonian dairy herds, each comprising ≥100 cows, via questionnaire, sample collection, and on-farm scoring and measurements. The final dataset included 147 questions divided into 13 meaningful blocks. The outcome variables were annual herd-level calf mortality risk during the first 21 days (MR21) and 22–90 days (MR90) using farm records and the national cattle database, respectively. The average MR21 was 5.9% (median 4.4%, range 0.0–26.8%) and the average MR90 was 2.7% (median 2.3%, range 0.0–12.7%). Of the 13 thematic variable blocks, the most important blocks explaining calf mortality were ‘Routine stress-inducing activities’, ‘Herd characteristics’, ‘Calving management’, ‘Calf housing during 5–21 days’, and ’External biosecurity’. The most influential single variables associated with higher overall calf on-farm mortality during the preweaning period were poorer cleanliness scores of calving animals and calves having access to an outdoor area during the first 21 days of life. Detected risk factors for MR21 were calf barn age > 20 years, allowing the calves to suckle the first colostrum, bucket feeding calves during the first three weeks, disbudding all calves (compared to only heifer calves), and disbudding at 21–29 days of age. Risk factors for MR90 included the use of automatic milk feeders and feeding waste milk during the first three weeks, early introduction of calves to large group pens and higher in-pen age differences, absence of forced ventilation during the first three weeks, opportunity for feces to spread between calf pens, and use of calving pens for sick animals. Washing and disinfection of newborn calves’ pens and testing colostrum quality were protective factors against both MR21 and MR90. Other protective practices for MR21 were related to proper colostrum feeding routines, whereas lower MR90 was mostly associated with efficient external biosecurity practices and vaccination programs. The multiblock model proved to be beneficial in providing a broader understanding of the importance of different management areas on calf mortality.

## Simple Summary

Calf mortality is a key indicator of calf health and welfare issues within the herd. Despite extensive research on the risk factors of calf mortality, its intricate nature continues to pose challenges for effective management. Previous studies have predominantly focused on individual risk factors, and information on the most influential management areas is lacking. In the present study, we address this issue by providing a more comprehensive overview of calf mortality risk factors to aid farmers, veterinarians, consultants, and other parties to prioritize farming areas, thus guiding them towards the most efficacious interventions.

## 1. Introduction

Each surviving calf increases farmers’ freedom to choose only the best calves as replacement animals, thus increasing the value of their herd. With the ongoing intensification of the dairy industry, characterized by increasing farm sizes, more complex farm structures, a greater reliance on paid labor, and the automation of many processes, the industry is undergoing significant transformation [1,2]. These changes highlight the need to establish farm conditions and routines that ensure the good health of animals, as any deviations affect a large number of animals simultaneously. An increase in herd size has also been linked to higher mortality [3,4,5,6,7], which is a recognized indicator of farm animal welfare [8]. Animal welfare is widely regarded as a priority by both stakeholders in the dairy industry and the lay public [9,10], especially considering the constant intensification of dairy production [1]. Under such circumstances, farmers could benefit from improving and maintaining the overall good health of the herd, which sets the grounds for the development and implementation of herd health programs.

Calf mortality arises from a variety of diseases and disorders, with diarrhea and respiratory diseases being the leading causes of morbidity and mortality during the preweaning period [11]. Septicemia, congenital anomalies, navel and joint infections, and trauma are less common causes of calf mortality, while up to 25% of deaths in young calves remain unexplained [12,13,14,15,16,17]. Because of its multifactorial etiology, calf mortality is inherently complex to study, yet identifying relevant risk factors is essential for improving calf health. Previous research has highlighted a range of animal- and herd-level factors associated with calf mortality. For example, mortality has consistently been higher in male calves, in cases of multiple births or dystocia, and among calves with lower serum immunoglobulin G (IgG) concentrations [18,19,20,21,22,23,24]. In contrast, while calf breed has been widely studied as a risk factor for mortality, findings remain inconsistent—some studies associate higher mortality with dairy [5,6,25] or beef [26,27] breeds in general, whereas others identify Holstein breed, being crossbred, or the absence of a predominant breed within a herd as risk factors [28,29,30,31]. Although research has predominantly focused on animal-level factors, a wide range of herd-level risk factors has also been reported, underscoring the complexity of management-related influences. Management practices such as navel disinfection in newborn calves [32], isolation of clinically diseased calves [33], or implementation of the all-in-all-out rearing system [30] have been associated with reduced mortality. The relationship between herd size and calf mortality has been investigated extensively over the past two decades, with most studies reporting higher mortality in larger herds [4,12,34,35,36,37,38], while some describe an opposite trend [24,32]. Other commonly reported herd-level risk factors include delayed colostrum feeding, cattle purchases, and higher disease incidence [3,24,31,39,40,41,42,43]. Many earlier studies have considered only a limited number of individual factors from various management areas [3,7,21,44,45,46] or included farms of markedly smaller or highly variable sizes [11,47,48], thereby limiting the applicability of their findings to large-scale dairy operations. In addition, a considerable proportion of research has been conducted under climatic conditions and production systems different from those in the Northern Hemisphere [49,50,51], which may restrict their broader applicability. Some studies have also assessed calf mortality across the entire preweaning period or have not clearly specified the age range under investigation [3,8,25,41,51,52,53]. However, the health issues and underlying risk factors vary substantially throughout the preweaning period [54]. Most importantly, the relative importance of different management areas remains insufficiently understood, despite their potentially diverse contributions to calf health outcomes.

Studies on the above-mentioned risk factors most often use generalized linear models, which have several advantages but also important limitations in large-scale, multifactorial analyses. Generalized linear models require modeling each outcome separately, are affected by multi-collinearity, and usually omit grouping explanatory variables into thematic blocks, making it difficult to evaluate the relative importance of different management areas [55]. To address this gap, the present study employs a novel statistical method in veterinary epidemiology: multiblock partial least squares (mbPLS) analysis [56,57]. This approach is particularly well-suited for investigating complex health issues described by numerous interrelated variables, which are often difficult to evaluate comprehensively within a single model. It enables the inclusion of a large number of variables organized into thematic blocks and demonstrates both the collective contribution of each block and the individual effect of each predictor variable on the outcome [55]. The outcome block itself can incorporate multiple variables that describe the same problem. Additionally, mbPLS offers greater stability in the presence of multicollinearity among explanatory variables—an advantage over many traditional methods [55]. Most importantly, the approach allows thematic blocks to be ranked according to their relative importance on the outcome. Applied to calf mortality, this helps to identify which management areas warrant the most attention—an insight not provided by previous studies on calf mortality risk factors. The aim of this study was to analyze a broad set of herd-level risk factors for calf mortality and to demonstrate the contribution of different management areas to calf mortality during the preweaning period using mbPLS analysis.

## 2. Materials and Methods

### 2.1. Herd Recruitment

The present study was part of a larger project aimed at including 120 Estonian dairy herds with at least 100 dairy cows to reveal the risk factors for dairy cow culling and cattle mortality. Additional inclusion criteria were a loose-housed keeping system for milking cows and no intention of terminating production in the coming years. A list of 182 herds meeting the size criterion was obtained from Estonian Livestock Performance Recording Ltd. in January 2019. In this study, a herd was defined as a unit(s) of dairy cows managed as one operation together with any associated youngstock unit(s). A single youngstock unit could be part of multiple herds if one owner had more than one milking farm but shared youngstock unit(s). A sample size of 120 herds was chosen based on the target population size, the expected number of herds compliant with the enrollment criteria and willing to participate in the study, and the available research resources. A random sample of 120 farms was taken from the list of all herds using a random number generator in Stata^®^ MP14.2 (College Station, TX, USA: StataCorp LP). Farms were then contacted individually by phone to ascertain compliance with the additional inclusion criteria, and to explain the objectives and methodology of the study. If a contacted herd did not meet the inclusion criteria or did not wish to participate in the study, a new herd was randomly selected from the sampling frame. A total of 169 herds were contacted before a final sample of 120 herds was obtained. All participating farms were visited by one of two veterinarians between August 2019 and July 2020.

### 2.2. On-Farm Data Collection

In this study, data were collected through interviews with farm representatives (farm manager or veterinarian) and through animal-based assessments and measurements performed on-site. To ensure clarity and consistency, the entire data collection procedure was practiced during theoretical training sessions and pretested in three supervised team visits. After each visit, discussion sessions were held to identify and resolve discrepancies, ensuring standardized data registration.

The questionnaire was developed by the authors in consultation with herd health veterinarians. It incorporated findings from previous research, expert insights from cattle health specialists, and feasible farm-level data, providing a comprehensive basis for assessing risk factors and management areas related to calf morbidity and mortality. The final version of the questionnaire consisted of 236 questions.

The on-farm assessments used in this study involved measuring the per-animal pen or cubicle area during calving, as well as the calf pen area within specific age ranges: the first 4 days, 5–21 days, and 22–90 days. In addition, cleanliness of the calving animals and calves aged 22–90 days was scored on a four-level scale: 0 = completely clean, 1 = slightly dirty, 2 = moderately dirty, and 3 = very dirty [58]. Two body regions were scored in calves: lower hind limbs and upper hind limbs together with flanks. For cows, the cleanliness of the udder area was recorded in addition to that of the aforementioned areas. Assessments were performed on 25% of the animals in the respective groups, with a minimum of 10 animals. In cases of inequality in the cleanliness of the right and left parts of the body, the dirtier side was chosen for assessment.

### 2.3. Sample Collection

To determine the herd status of the selected infectious diseases, a bulk tank milk (BTM) sample was collected from each milk tank with milk available during the herd visit. Ten blood samples were also collected from 8- to 16-month-old heifers, selected randomly from each group pen including the targeted age group. These samples were tested for bovine respiratory syncytial virus (BRSV), bovine herpesvirus 1 (BHV-1), bovine viral diarrhea virus (BVDV), *Mycobacterium avium* spp. *paratuberculosis*, *Mycoplasma bovis*, and *Salmonella* Dublin antibodies. Considering the sample size, laboratory test characteristics and the number of 8- to 16-month-old heifers in the study farms, the minimal detected disease prevalence ranged from 22% to 53%, varying between pathogens. Specific details about the justification of the sample size and sample collection procedures, along with the detected between-herd and within-herd prevalences, can be found in Mõtus et al. [59].

From each study farm, 5–15 blood samples were obtained from calves up to 7 days old, and up to 5 fecal samples from calves up to 21 days old exhibiting clinical signs of diarrhea. Blood samples were drawn from the jugular vein using 9 mL vacuum tubes and 20 G vacutainer needles to measure serum IgG concentration. Fecal samples were collected either directly from the rectum or during defecation to detect the presence of common causative agents of diarrhea. Each calf was sampled once. In farms where five fecal samples or five calf blood samples could not be obtained due to an insufficient number of calves within the suitable age range and/or diarrheic (n = 66 and n = 36, respectively), additional sample collection visit was performed between December 2020 and January 2021. During the second sampling visit, diarrheic calves were preferred for fecal sampling; however, if an insufficient number of such calves were present, samples were collected from apparently non-diarrheic calves within the determined age range. In total, five fecal samples and five calf blood samples could not be obtained in the two sampling rounds from five and two farms, respectively. On average, five fecal samples (range 0–8 samples) and nine calf blood samples (range 3–17 samples) were collected per herd.

### 2.4. Laboratory Analyses and Data Interpretation

All collected samples were refrigerated at 4 °C and transported to the laboratory. Blood samples from calves and heifers, as well as BTM samples, were centrifuged at 3500 rpm for 8 min. The defatted milk and separated serum samples were frozen at −20 °C and stored until analysis. The fecal samples were immediately frozen until analysis. Sample pre-treatment and all ELISA procedures were carried out according to the manufacturer’s instructions. ELISA plates were incubated under controlled conditions (Melag Incubat 80/85), and absorbance was measured with a Sunrise™ Tecan spectrophotometer (Tecan Group Ltd., in Männedorf, Switzerland) (Magellan software, 405–620 nm). Details about the diagnostic tests used for BTM, heifer serum sample and calf fecal sample analyses can be found in Supplementary Table S1.

Herd status regarding BHV-1, BRSV, BVDV, *Mycobacterium avium* spp. *paratuberculosis*, *Mycoplasma bovis*, and *Salmonella* Dublin was established according to the antibody testing results of BTM and heifer blood samples, as described in Mõtus et al. [59] using the commercial ELISA tests. Herds that reported vaccinating their cattle against BHV-1, BVDV, or BRSV were categorized separately in the models. Due to the exclusive availability of BHV-1 marker vaccines, a glycoprotein-E ELISA was used for testing BHV-1 antibodies in herds that were currently or had previously vaccinated their animals with BHV-1 glycoprotein-E deleted marker vaccines. This approach enabled differentiation between vaccinated and infected animals. While vaccinated animals test negative for the deleted portion of the virus, infected animals also test positive for glycoprotein-E due to exposure to the wild-type virus.

Calf fecal samples were tested for the presence of bovine rotavirus, bovine coronavirus, enterotoxigenic *Escherichia coli* and *Cryptosporidium parvum* antigens using an antigen ELISA kit BIO K 348 (Bio-X Diagnostics, Rochefort, Belgium), and the results were used as a binary variable, indicating the presence or absence of the respective pathogen among the samples collected from the herd.

Calf serum IgG content was measured using a MonoScreen QuantELISA Immunoglobulin Easy kit (BIO K 420; Bio-X Diagnostics, Rochefort, Belgium), with intra- and inter-assay variations of <10%.

### 2.5. Mortality Calculations and Farm-Level Data

In Estonia, calves must be ear-tagged within the first 20 days of life [60]; therefore, the registry data may have missed some calf mortality cases which occurred during the first three weeks of age, before ear-tagging. To calculate the annual calf mortality risk during the first 21 days of life (hereafter, the younger age group [YAG]), data on the number of live births and occurred deaths (including unassisted deaths and euthanasia) were gathered from farm records. Similar data for calves aged 22–90 days (hereafter, the older age group [OAG]) were obtained from the Estonian Agricultural Registers and Information Board. For each herd, annual calf mortality risk was calculated over the one-year period preceding the farm visit—MR21 for the younger age group and MR90 for the older age group (Equations (1) and (2), respectively).

(1) $$annual\;calf\;mortality\;risk\left(YAG\right)=\frac{yearly\;number\;of\;calves\;died\;before\;22\;during\;one\;year}{yearly\;number\;of\;live-born\;calves\;during\;the\;same\;year}\times 100$$

(2) $$\mathrm{annual}\;\mathrm{calf}\;\mathrm{mortality}\;\mathrm{risk}\;\left(\mathrm{OAG}\right)=\frac{\mathrm{yearly}\;\mathrm{number}\;\mathrm{of}\;\mathrm{calves}\;\mathrm{died}\;\mathrm{between}\;22-90\;days\;of\;age\;during\;one\;year}{\mathrm{yearly}\;\mathrm{number}\;\mathrm{of}\;\mathrm{calves}\;\mathrm{present}\;\mathrm{in}\;\mathrm{the}\;\mathrm{farm}\;\mathrm{at}\;22–90\;\mathrm{days}\;\mathrm{of}\;\mathrm{age}\;\mathrm{during}\;\mathrm{the}\;\mathrm{same}\;\mathrm{year}\;}\;\times \;100$$

For each herd, data regarding the number of cows and the herd’s current average milk yield (on the date of the visit and exactly one year before it) were collected from Estonian Livestock Performance Recording Ltd. The average number of cows and the average 305-day milk yield were calculated as the average of the appropriate number on the day of the farm visit and one year before the visit.

### 2.6. Data Editing

The questionnaires were digitalized using an electronic survey tool LimeSurvey [61], exported to an Excel spreadsheet, and combined with data regarding the number of births, deaths, mortality risk of the YAG and OAG, herd average number of cows, and average milk yield. Questionnaire data were screened, and continuous variables with non-normal distribution were categorized at their quartile or median values to enable the use of the multiblock model, which relies on linear associations, and to address instances where some variables were not applicable to certain herds. For categorical variables, some answer categories were merged, if possible, to avoid categories that entailed a small number of observations. The proportion of dirty animals for each assessed body region was calculated by summing the number of animals with moderately dirty or very dirty cleanliness (score 3 or 4) in their respective area, dividing the sum by the number of assessed animals, and presented as a percentage. For each herd, the average IgG concentration of tested calves was calculated as the arithmetic mean of their blood sample results.

Based on consultations with herd health experts, all collected data concerning calves were consolidated into 13 meaningful categories, including a total of 121 questionnaire variables, 11 variables based on farm measurements, 5 variables derived from animal scoring results, herd average IgG level in calves’ sera, and variables describing herd status for the 10 tested pathogens (Supplementary Tables S2 and S3). Two herds were eventually excluded from the analyses because they transported their calves to another farm after the colostrum period. Therefore, the data from 118 herds were included in this study

### 2.7. Statistical Analysis

All explanatory variables were initially screened for their univariable associations with both outcome variables (MR21 and MR90) using negative binomial regression analysis; however, the two blocks entailing questions about calf feeding and housing management during 22–90 days of age were not represented in the YAG univariable analyses because of the unequivocal lack of possible association with MR21. A causal diagram was developed prior to the analyses, hypothesizing that herd size could act as a biological confounder. Consequently, herd size, treated as a continuous variable, was included as a fixed effect in the univariable model to control for its confounding effect. All variables associated with at least one outcome variable at a *p*-value ≤ 0.2 [62] were included in subsequent analyses.

To detect and assess the strength of collinearity between the preselected variables, a variance inflation factor was used with a cutoff value of 5 indicating substantial collinearity [62]. For variables exceeding the threshold value, the collinear variable with the lowest significance to the outcome variables was removed. All categorical explanatory variables were then converted to dummy variables, and the outcome variables were log-transformed. The mbPLS analysis was applied subsequently to study how each thematic block contributes to the prediction of the combined preweaning calf losses (the Y block consisting of MR21 and MR90), and to identify, within each thematic block, the variables that significantly contribute to MR21, MR90, and the Y block [63]. In the mbPLS analysis, one or more outcome variables are incorporated into the Y block and are explained by K explanatory variable blocks (X1, …, XK). Briefly, as explained by Bougeard et al. [55], the principle of the model is that each block of variables is included as a separate dataset and summed by a latent variable (component) which is a linear combination of all the variables inside the dataset. This allows the use of multiple datasets, making it more resilient to multicollinearity within the blocks. The method develops a global component which is related to all the explanatory variables included in different datasets, sums up the partial components associated with each of the blocks (datasets) and is also closely related to the linear combination of the variables in the outcome block Y. Two recent studies in the field of veterinary epidemiology also explain the usage and mechanism of the mbPLS method [64,65].

The model was run with centered and scaled data, and all blocks were assigned equal weights regardless of the number of variables in the block. After the initial mbPLS model, variables or categories that were not associated with either the MR21, MR90, or Y block were removed to construct the final mbPLS model. The mbPLS results were interpreted using a model with two components and described based on the “variable importance index” and “block importance index,” which represent the relative contribution of each explanatory variable and each explanatory block in explaining preweaning calf mortality estimates in the Y block, respectively [55]. Based on 500 repetitions of the bootstrap simulation, 95% confidence intervals (CI) were calculated for the average contribution estimates of each block, variable, and variable category. If the calculated 95% CI did not include zero, the respective block, variable, or category was considered to be significantly associated with the outcome.

Negative binomial regression analyses were performed with Stata^®^ MP14.2 (College Station, TX, USA: StataCorp LP) and for mbPLS, R statistical software version 4.2.3 [66] and R package ade4 [63] was used.

## 3. Results

### 3.1. General Characteristics

**Figure 1:**
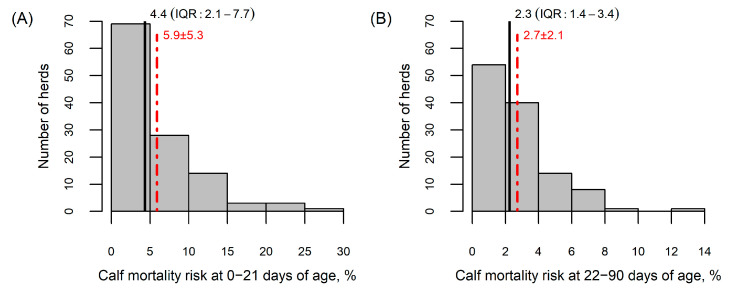
Distribution of herds according to the annual calf mortality risk: (**A**) during the first 21 days of age; (**B**) during 22–90 days of age. The red dotted line indicates the mean value and red numbers represent the mean ± standard deviation. The black line shows the median and black numbers represent the median and interquartile range (IQR).

The average number of cows in the 118 study farms was 518 (range 92–2275) and the average 305-day milk yield per cow was 10,311 kg (range 5983–13,155). The average number of calves born per year was 536 (range 90–2715). The average annual herd-level calf mortality risk was 5.9% (median 4.4, range 0.0–26.8) during the first 21 days of age and 2.7% (median 2.3, range 0.0–12.7) during 22–90 days of age (Figure 1).

### 3.2. Importance of Farming Areas and Individual Risk Factors in Explaining Overall Preweaning Calf Losses

Following the screening of variables using negative binomial regression analysis, 33 and 62 variables were included in the multiblock analysis for the YAG and OAG, respectively (Table 1). Complete results of the univariable analyses are presented in Supplementary Tables S2 and S3.

**Figure 2:**
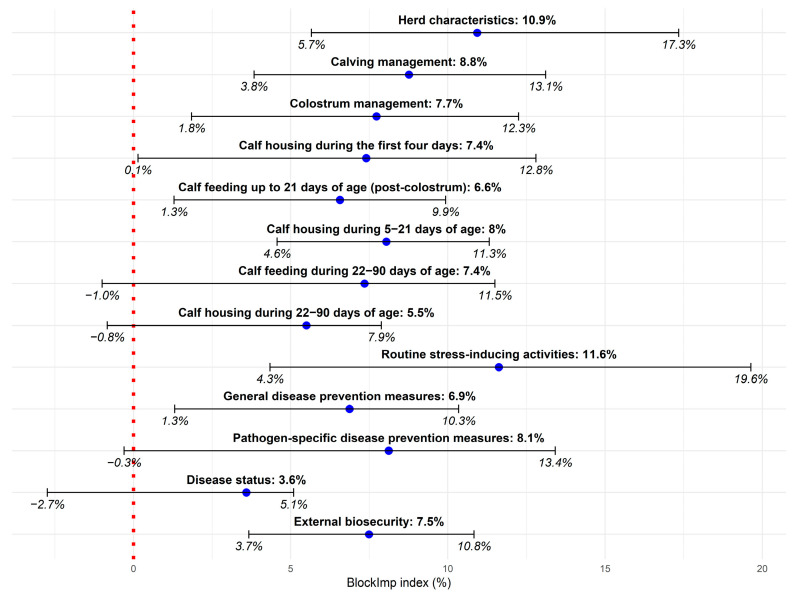
Block importance index for the 13 thematic blocks of variables explaining preweaning calf mortality expressed through mortality up to 21 days of age and mortality during 22–90 days of age. Blue points represent observed block importance values, horizontal lines indicate 95% confidence intervals, and the dotted red line marks the zero reference level.

**Figure 3:**
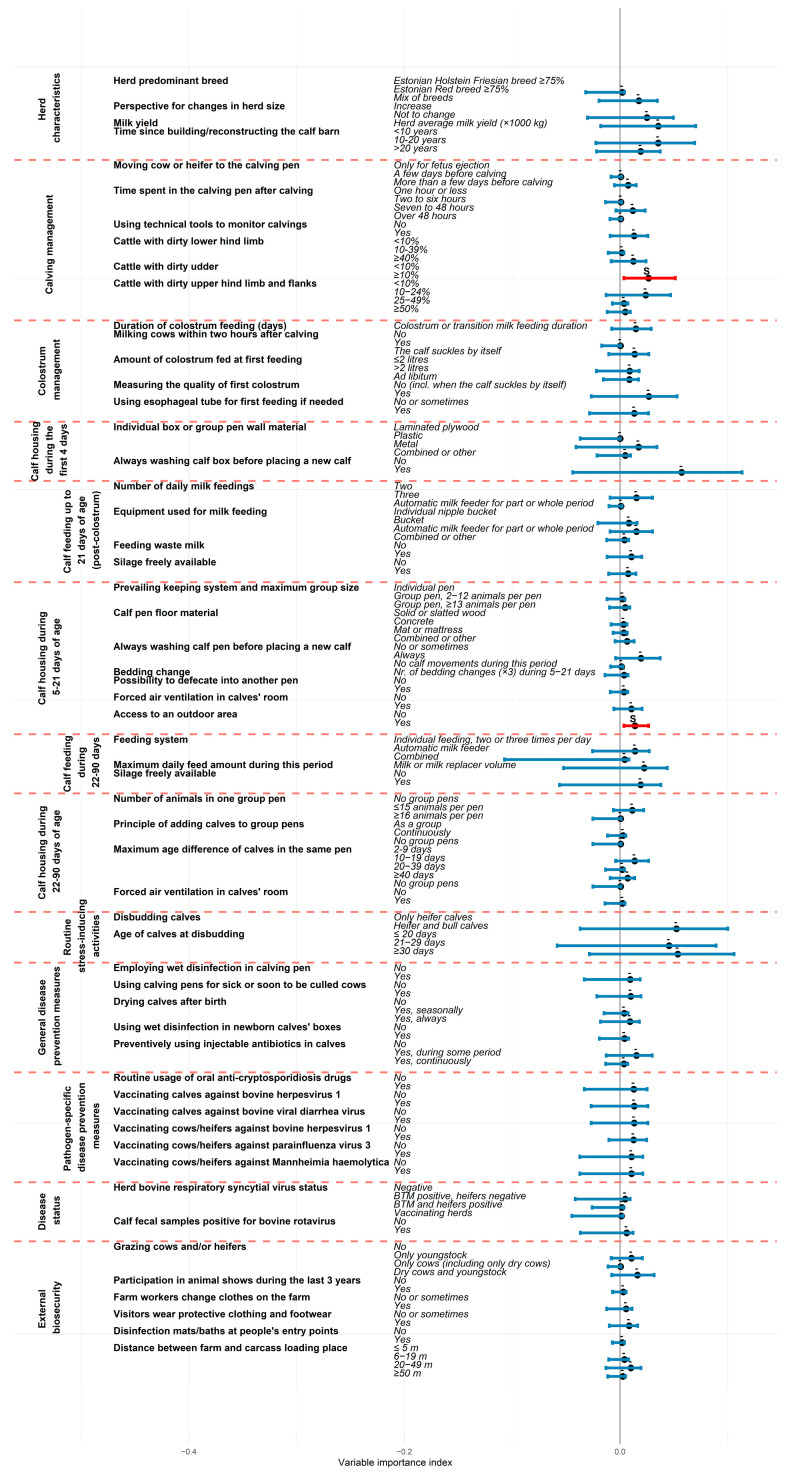
The variable importance index of the preselected predictor variables in explaining the composite outcome Y reflecting preweaning calf mortality (based on two fundamental variables of mortality up to 21 days of age and mortality during 22–90 days of age), according to multiblock partial least squares analysis. The red color of the 95% confidence interval, together with ‘S’ above the line, indicates a statistically significant difference compared to the first variable category.

Overall, the mbPLS model explained 67% of the total variability in herd calf mortality risk (the Y block including MR21 and MR90). Nine out of the thirteen analyzed thematic blocks contributed significantly to explaining preweaning calf mortality. According to the mbPLS, the most influential variable blocks were “Routine stress-inducing activities” (block importance index (BlockImp) = 11.6%, 95% CI 4.3; 19.6), “Herd characteristics” (BlockImp = 10.9%, 95% CI 5.7; 17.3), “Calving management” (BlockImp = 8.8%, 95% CI 3.8; 13.1), “Calf housing during 5–21 days of age” (BlockImp = 8.0%, 95% CI 4.6; 11.3), and “External biosecurity” (BlockImp = 7.5%, 95% CI 3.7; 10.8) (Figure 2). Two variables were found to have a significant contribution to explaining calf losses (Y block) in this study: “Proportion of animals with dirty udder in the calving group” (variable importance index (VarImp) = 2.7%, 95% CI 0.4; 5.1) and “Having access to outdoor area during 5–21 days of age” (VarImp = 1.4%, 95% CI 0.4; 2.6) (Figure 3).

### 3.3. Risk Factors for Individual Calf Mortality Estimates

Higher milk yield was a protective factor against MR90 (regression coefficient β = −0.54, 95% CI −1.02; −0.31). Calf barn construction or the last renovation time more than 20 years ago was identified as a risk factor for MR21 (β = 0.40, 95% CI 0.14; 0.79, compared with <10 years), whereas barns renovated or constructed 10–20 years ago showed a protective effect (β = −0.51, 95% CI −0.87; −0.20).

Both MR21 and MR90 were lower on farms where the quality of the first colostrum was always measured before feeding (β = −0.30, 95% CI −0.64; −0.05 and β = −0.40, 95% CI −0.74; −0.19, respectively). Extended duration of colostrum feeding and the usage of an esophageal tube to administer the first colostrum when adequate ingestion could not be confirmed were both protective against MR21 (β = −0.32, 95% CI −0.60; −0.05 and β = −0.30, 95% CI −0.67; −0.06, respectively). Allowing the calves to suckle the first colostrum meal and bucket-feeding calves during the first 21 days were practices associated with a higher MR21 (β = 0.29, 95% CI 0.09; 0.62 and β = 0.35, 95% CI 0.04; 0.64, respectively), compared to feeding with individual nipple bucket. Feeding waste milk from cows under antibiotic treatment or in a withdrawal period to calves during the first three weeks of life was associated with increased MR90 (β = 0.40, 95% CI 0.18; 0.74). Higher MR90 was also observed in herds where calves were fed via automatic milk feeders during the first 21 days (β = 0.28, 95% CI 0.07; 0.60), compared to feeding with individual nipple bucket.

MR90 was higher in herds where calves were kept in group pens containing 13 or more animals during their first three weeks of life as opposed to individual housing (β = 0.23, 95% CI 0.02; 0.49). MR90 was also elevated in herds where calves could defecate into another calf’s pen (β = 0.19, 95% CI 0.02; 0.45). A maximum age difference of 20–39 days among calves in the same group pen was associated with increased MR90 (β = 0.20, 95% CI 0.02; 0.50, compared with an age difference of 2–9 days), whereas forced ventilation during the first three weeks of life was associated with lower MR90 (β = −0.33, 95% CI −0.59; −0.14).

Disbudding all calves and disbudding at 21–29 days of age were both associated with a higher MR21 (β = 0.62, 95% CI 0.23; 1.06, compared to disbudding only heifer calves; and β = 0.58, 95% CI 0.26; 1.07, compared to disbudding at ≤20 days, respectively). In contrast, disbudding at ≥30 days of age was protective against increased MR21 (β = −0.58, 95% CI −1.16; −0.29, compared to disbudding at ≤20 days).

**Figure 4:**
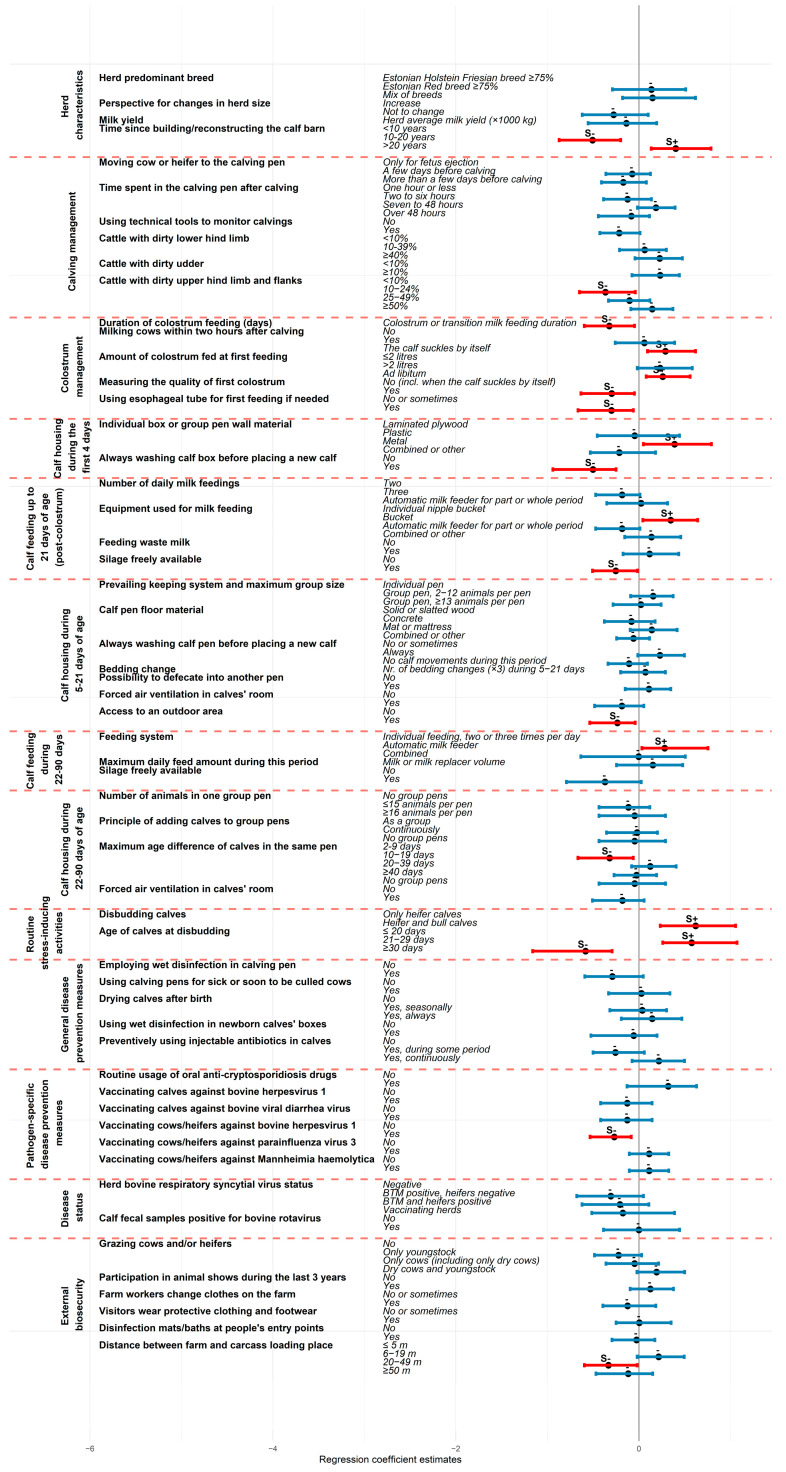
Association between the preselected predictor variables and annual calf mortality risk during the first 21 days of age according to multiblock partial least squares regression analysis. The regression coefficient estimates are presented as dots and the 95% confidence intervals are illustrated with bold horizontal lines with a red color indicating statistically significant difference compared to the first variable category. S+ above the line refers to a statistically significant increase and S− indicates a decreased effect on calf mortality during the first 21 days of age.

**Figure 5:**
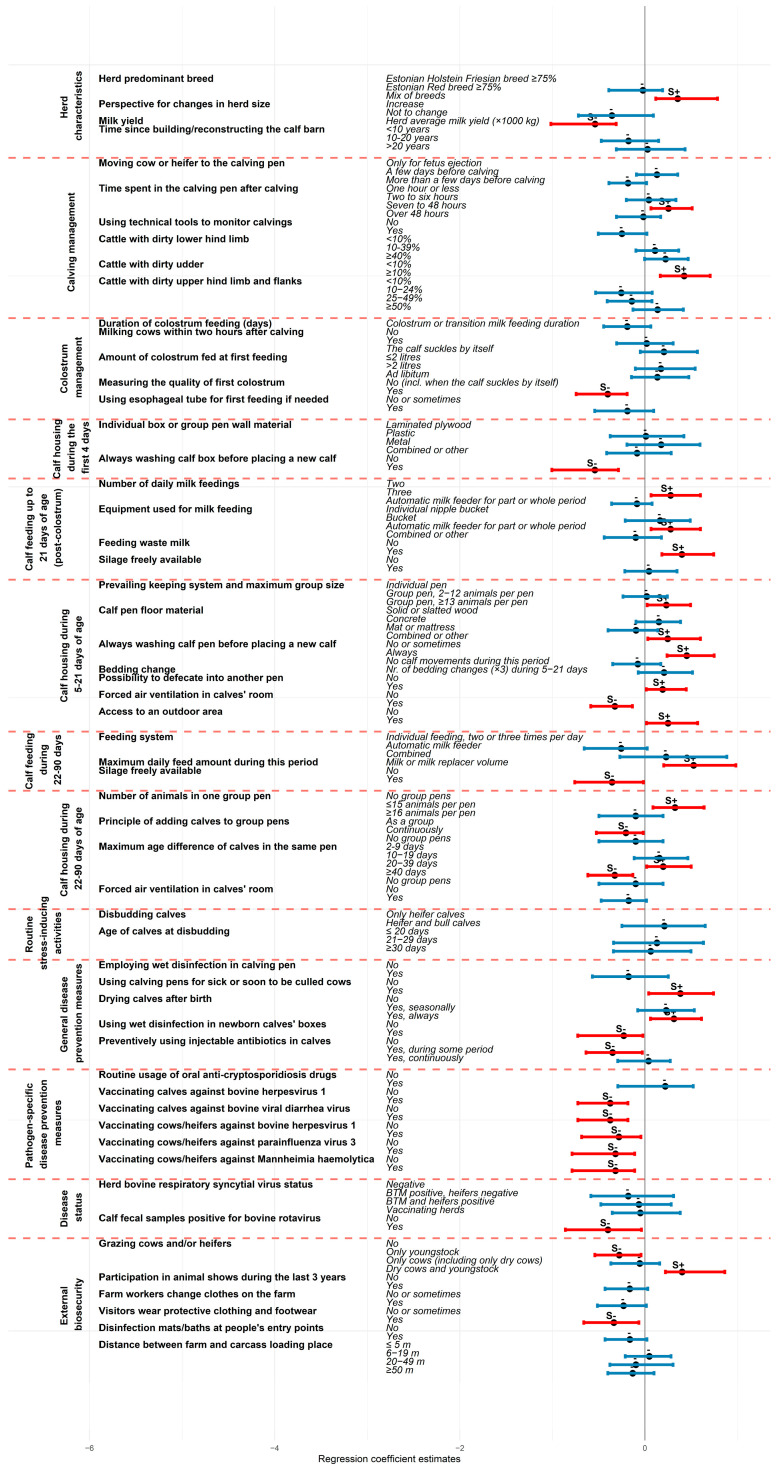
Association between the preselected predictor variables and annual calf mortality risk during 22–90 days of age according to multiblock partial least squares regression analysis. The regression coefficient estimates are presented as dots, and the 95% confidence intervals are illustrated with bold horizontal lines with a red color indicating statistically significant difference compared to the first variable category. S+ above the line refers to a statistically significant increase and S− indicates a decreased effect on calf mortality during 22–90 days of age.

Always cleaning and washing the pen before introducing a new calf was identified as an effective protective measure against both MR21 (β = −0.50, 95% CI −0.94; −0.25) and MR90 (β = −0.54, 95% CI −1.01; −0.29). Consistent use of wet disinfection in newborn calves’ pens between introductions was associated with reduced MR90 (β = −0.23, 95% CI −0.73; −0.02). Conversely, using calving pens for sick or soon-to-be culled cows was associated with higher MR90 (β = 0.38, 95% CI 0.04; 0.74). A lower MR90 was observed in farms where visitors interacting with animals always wore protective or farm-specific clothing and footwear (β = −0.34, 95% CI −0.66; −0.07), in farms where youngstock were grazed (β = −0.28, 95% CI −0.54; −0.04, compared to not grazing any animals), or when calf fecal samples tested positive for bovine rotavirus (β = −0.40, 95% CI −0.86; −0.04). MR21 was also lower on farms where cows were vaccinated against BHV-1 (β = −0.27, 95% CI −0.54; −0.08). Vaccination of calves against BHV-1 and bovine viral diarrhea virus (β = −0.38, 95% CI −0.73; −0.18 on both cases), and vaccination of cows against BHV-1, parainfluenza-3 virus, and *Mannheimia haemolytica* were also protective against MR90 (β = −0.28, 95% CI −0.68; −0.04 for BHV-1, β = −0.32, 95% CI −0.79; −0.11 for the others). The full mbPLS results are provided in Supplementary Table S4 and illustrated in Figure 4 and Figure 5.

## 4. Discussion

To the best of our knowledge, this study represents the first analysis of herd-level risk factors for calf mortality using multiblock partial least squares analysis. The multiblock method provides a broader perspective on the critical aspects of calf management by indicating the significance of thematic blocks and highlighting the individual variables influencing calf mortality. Preweaning calf mortality is a multifactorial outcome, and our model was able to explain 67% of its variability, capturing a substantial share of the herd-level influences contributing to these losses.

### 4.1. Importance of Farming Areas and Individual Risk Factors in Explaining Preweaning Calf Losses

One of the most influential thematic blocks explaining preweaning calf losses combined information about calf housing conditions from five days to three weeks of age. Although this block included questions on different aspects of calf housing, these questions more or less portrayed internal biosecurity during this critical period. Infectious diseases pose the greatest risk to calves during the first weeks of life, prior to the maturation and full activation of their immune system [67,68]. The external biosecurity block was also among the most influential ones, affirming the importance of infectious disease prevention in tackling calf mortality. Conversely, among all herd disease status variables, only the presence of rotavirus infection in at least one tested calf was significantly associated with lower calf losses on the study farms. This obscure association might derive from the sampling and data handling methods. Since samples were collected only once, the results rather reflected the current epidemiological situation on the farm than the entire preceding year. Additionally, the relatively small number of calf fecal samples obtained may not have adequately described the prevalence of these pathogens, nor did it allow for the analysis of the impact of pathogen prevalence on calf mortality.

The block describing general herd characteristics exhibited a notable block importance although none of its variables were significantly linked to calf losses. Most likely, the questions in this thematic block disclose broader insights into the farms and reflect on their perspectives, innovativeness, and overall mentality, thereby elucidating its high importance. The block describing routine stress-inducing activities was also highly important, although the questions included in the final model only described disbudding practices on the farm. Disbudding causes substantial tissue damage in calves, significantly affecting their long-term well-being [69]. The timing of this stressor appears to have a broad impact on the health and vitality of calves.

Single variables significantly contributing to increased calf mortality during the preweaning period were ≥10% of cows having dirty udder area in the calving pen and calves having access to an outdoor area during the first 21 days of their life. Inferior cow cleanliness scores represent the overall hygiene of the barn environment and have been linked to inferior health and increased mortality of cows [65,70]. Poorer hygiene and increased manure contamination in the calving pen have also been shown to increase the risk of pathogen transmission to newborn calves [71,72], and could possibly lead to long-term adverse health effects. Only a few studies have been conducted on the effects of outdoor area access on calf mortality. One study conducted on veal calves in Switzerland reported that access to an outside pen was a significant risk factor of calf mortality [3], whereas another Swiss study demonstrated that access to an outdoor pen was associated with a higher incidence of antimicrobial treatment [73]. Outside areas are not meant for grazing purposes but are usually smaller paddocks to which the calves have year-round access. Although not registered in our questionnaire, these are usually soil-bottomed, making them difficult to clean and disinfect, possibly leading to a higher pathogen load in the area. Access to an outdoor area was also associated with larger group size, which could contribute to higher mortality by increasing the risk of disease occurrence [74]. However, as this practice was apparent in only six herds, we are cautious in drawing definitive conclusions based on this finding.

### 4.2. Risk Factors for Individual Calf Mortality Estimates

Two significant variables were detected in the herd characteristics block: average milk yield and calf barn age. Similarly to previous studies [33,75], higher milk yield was identified as a protective factor against MR90. The average milk yield in our study farms exceeded 10,000 kg per cow and such a high production level necessitates the implementation of premeditated activities and thoughtful routines in all farming areas, possibly including calf rearing. Consistent management and entrenched routines may lead to both better animal health as well as enhanced production results. Calf barn construction or the latest renovation being over 20 years ago was determined to be a risk factor for MR21, while a barn age of 10–20 years was protective against increased mortality in the YAG. In Estonia, the older barns used for rearing youngstock are often renovated former cow tie-stall barns from the Soviet era, which do not meet the needs of current farm sizes and animal husbandry peculiarities, often showing problems such as inadequate ventilation and an unsuitable air microclimate. Insufficient air quality has been linked to increased calf morbidity and mortality previously [76]. The importance of effective ventilation was also demonstrated in this study, as farms that housed their calves in rooms equipped with forced ventilation during the first weeks of life had a lower MR90.

It has long been recognized that calves’ survival and resilience to infections depend on passive immunity acquired from colostrum and milk [77,78]; as such, ensuring that calves receive an adequate quantity of high-quality colostrum shortly after birth is an integral aspect of neonatal calf management. We found that both MR21 and MR90 were lower on farms where the quality of the first colostrum was always measured before feeding. Optimal colostrum quality is pivotal in ensuring sufficient intake of IgG by the calf in order to avoid the development of failure of passive transfer of immunity (FPTI), a condition associated with significantly elevated morbidity and mortality risks in calves [23]. Interestingly, in the present study, the farm-level average IgG content in calf serum was not associated with mortality. This may be due to the single sampling of calves, which reflects only short-term colostrum feeding practices. A longer duration of colostrum feeding was another protective factor against MR21. Longer colostrum or transition milk provision decreases the morbidity and mortality in calves through continuous supply of antibodies via lactogenic immunity [33,79]. Allowing the calf to suckle the first colostrum was identified as a risk factor for MR21 in this study, although previous research has shown variable effects of this practice [12,45,74,80]. Despite the possibility of visually confirming suckling, allowing the calf to consume its initial colostrum meal independently precludes the assessment of both the quality and quantity of colostrum ingested. Consequently, this practice increases the risk of FPTI and contributes to increased morbidity and mortality in calves [23,80,81,82]. Using an esophageal tube for feeding the first colostrum in situations where confirmation of adequate ingestion is not feasible may reduce the incidence of FPTI and was identified as a protective measure for MR21 in the present study. Previous studies have also indicated that intervention with colostrum consumption in critical situations lowers the occurrence of FPTI and calf mortality [83,84].

Feeding calves with waste milk from cows undergoing antibiotic treatment or in a withdrawal period was associated with an increased MR90. Milk from cows under treatment exhibit higher bacterial counts, increased somatic cell counts, and antimicrobial drug residues [85,86,87]. Previous studies have yielded somewhat conflicting results regarding the effect of waste milk feeding on calf production parameters [85,86,88,89]. Still, it has been confirmed that feeding such milk increases diarrhea occurrence, alters calf fecal microbiota composition, and enhances the proportion and shedding of antimicrobial-resistant bacteria [46,85,86,87,90]. We also found that MR90 was higher in herds in which calves were fed via automatic milk feeders during the first three weeks of age. Previous studies have identified substantial differences in the adaptation time with automated feeders in calves that were introduced to this feeding system in early life [91,92]. Longer adaptation time was associated with a lower overall milk intake over the next few weeks in these studies. Milk intake, as well as the interval between feeder visits and drinking speed, has been linked to disease incidence in calves kept in automated milk feeder systems [93]. During the first 21 days of life, the main energy source for calves is milk or milk replacer, and a reduction in the amount of obtained nutrients and energy can possibly result in a deteriorated health status. Automatic milk feeders have also been associated with larger group sizes and greater within-pen age differences among calves [94], and both of these features were related to calf mortality in this study. Higher within-pen age difference among calves may enhance pathogen transmission between calves and was identified as another risk factor for MR90 in the present study, although previous research has not reached an agreement about this [41,95]. Housing calves in large group pens was found to be a risk factor for MR90 regardless of the duration of implementation and whether this was performed for male, female, or both sexes of calves. Early introduction to group pens has been linked to higher mortality [12], presumably due to the higher pathogen load and disease incidence in the group pen setting. Previous studies have shown that larger group sizes correlate with a higher incidence of respiratory diseases, a more severe and earlier onset of diarrhea, and reduced growth rates [74,96,97].

Increased MR90 was observed in farms where calving pens were used for sick or soon-to-be-culled animals. This is in line with previous research where using calving pens for other purposes, for example, for housing sick animals correlated with increased disease prevalence, including respiratory and digestive disorders [14,98]. Increased MR90 was also linked to the possibility of calves to defecate into neighboring pens. Lower contamination of the calves’ environment with feces from other calves has been linked to lower bacterial counts and better visible cleanliness [46] and signifies the importance of limiting horizontal pathogen transmission.

Disbudding all calves (compared to only heifer calves) and disbudding at 21–29 days of age were both identified as significant risk factors for MR21 identified from the thematic block “Routine stress-inducing activities”. Disbudding calves at ≥30 days of age was protective for MR21 compared to early disbudding. Bull calves are most likely disbudded only in herds where they are also reared until slaughter, and as male sex itself is a risk factor for mortality [5,19,29], we might assume that the higher mortality identified in herds where all calves were disbudded is probably derived from the higher proportion of bull calves in the herd. In an Estonian dairy herd setting, many changes in the housing and feeding of calves occur at around two to three weeks of age. This is the period at which calves are moved from individual housing to group pens, coinciding with changes in the calves’ environment, feed, and feeding systems, while also presenting challenges for calves as they adapt to increased competition and socialization. Ultimately, these changes entail high stress level for calves and additional stress from disbudding might be unbearable for young calves, resulting in higher mortality. The protective effect of a later disbudding age suggests that dispersing stressors over a longer period of time could be beneficial for calf health.

Disease control is undoubtedly one of the most perplexing challenges in a dairy farm and multiple influential variables were detected in the external biosecurity block. For example, a lower MR90 occurred on farms which always required a change of attire from visitors who come into contact with animals. Numerous variables related to the vaccination of calves and adult animals were also associated with lower calf mortality in this study. Previous studies have reported somewhat conflicting results regarding the effects of herd vaccination policies on calf mortality. Nevertheless, these studies are not directly comparable because of variability in factors such as herd size, management style, timing and route of the vaccination, included pathogens, or type of the vaccine [44,99,100,101]. The importance of limiting pathogen circulation was also implicated by questions from other thematic blocks, such as calving management, calf housing and calf feeding. Similarly to the results of other studies, maintaining good environmental hygiene in calf barns was associated with lower mortality risk in both age groups. Previous studies have demonstrated that cleaning and disinfecting calf pens leads to lower bacterial load and decreased mortality [30,46,102]. Calf pens were currently routinely cleaned and disinfected only in approximately 25% and 50% of farms, respectively, thus the importance of good environmental hygiene in calf barns should be stressed. The large number of significant variables related to biosecurity measures emphasizes the need to prevent the introduction and spread of infectious diseases on farms and provide calves with additional protection against infections.

### 4.3. Study Limitations

The limitations of this study should be considered when interpreting the results. Mortality estimates can be computed in a number of ways, and the calculation methods may substantially affect the obtained result [103]. Although some calves born alive who die before eartagging may appear as stillbirths in the national registry, this information is usually accurate in the farm’s own records. Therefore, the number of calves born alive and dying within the first three weeks of life (prior to routine eartagging) was determined from the farm records. Still, as we do not know the exact definition of stillbirth used on each farm, the mortality rate in the younger age group might be slightly underestimated, and some inaccuracies may have been introduced. However, we believe that registration behavior is not dependent on the mortality level of the farm; therefore, this possible bias is presumably random and does not influence the analyses of risk factors. It is important to note that direct comparisons of our mortality estimates with those reported in other studies may also be limited by inherent differences in study design and context. These include variations in age category margins, climatic conditions, and management practices—all of which can significantly influence calf mortality.

Most of the risk factors and mortality data used in this study were collected for the same timeframe, making it difficult to draw causal inferences. In addition, we are not aware of how long the recorded management practices were employed on the farms. While most of the questions in the questionnaire reflected predominant routines and practices implemented over the last year, animal scoring and measurements provided insights into the present conditions on the farm. Despite their potentially fluctuating nature, which may limit the ability of these parameters to represent an entire year-long observation period, we considered the information obtained from these variables important in explaining the complex nature of calf mortality. Animal-based scores summarize the interactions between numerous farm-based variables that are difficult to measure individually. For example, animal cleanliness reflects factors such as population density, housing type, indoor climate, bedding quantity, and frequency of bedding renewal or manure removal. Because herds were visited in different seasons within a one-year period, climatic parameters could not be included in the model, despite their recognized importance for calf mortality [6,25,50,76,104]. Nevertheless, some animal-based parameters in our dataset are likely to partially reflect the impact of indoor climate. Additionally, certain management practices identified as risk factors (e.g., housing system, use of forced ventilation, age of the barn) may affect mortality by shaping indoor climate conditions. Still, the exclusion of climatic variables may have limited the model’s ability to fully capture environment-related effects.

The one-time sample collection limited the information obtained from pathogen testing, as the results likely reflected the current epidemiological situation rather than the patterns of pathogen circulation over the entire preceding year. Additionally, although low pathogen prevalences were not a primary focus of this study due to their unlikely impact on herd health, the chosen sampling procedure may have underestimated the overall impact of pathogens on calf health and associated mortality. Nevertheless, the study aimed to account for the role of infectious diseases, which are known to be major contributors to young calf mortality [40,105,106], thereby enhancing the overall comprehensiveness of the resulting overview of calf mortality risk factors.

The multiblock model provided an extra dimension of information regarding the most important risk factor areas for calf mortality; however, the method also inflicted some restrictions on the interpretation of the results. First, all the variable blocks were attributed equal weights in the model, regardless of the varying number of variables contained in them. This may result in a higher importance of variables from blocks that contain fewer variables compared to variables from blocks with more variables. In addition, the allocation of some variables to specific thematic blocks was somewhat subjective. Finally, the multiblock partial least squares analysis cannot adjust for confounding effects. Although herd size was hypothesized as a confounder based on the causal diagram, the negative binomial regression model did not indicate a statistically significant confounding effect. Nevertheless, to ensure that only variables with an effect independent of herd size were included in the mbPLS model, herd size was controlled for during the variable selection stage.

## 5. Conclusions

This study used multiblock partial least squares analysis to identify herd-level risk factors and risk factor groups associated with preweaning calf mortality in large-scale dairy herds. This method allowed the inclusion of an extensive number of risk factors by grouping them into thematic domains and assessing their overall importance in relation to calf mortality, which, as the outcome variable, comprised mortality in two different age categories. The final model explained 67% of the total variability in calf mortality. The most influential management areas included calving management, early-life housing conditions, timing of stress-inducing procedures, and external biosecurity. At the variable level, poor udder cleanliness at calving and early outdoor access for calves were associated with higher mortality, whereas effective colostrum management, delayed disbudding, and consistent pen hygiene were protective. These findings suggest that the first three weeks of life may be a particularly critical period for calf survival within the preweaning phase. Prioritizing calving hygiene, optimizing colostrum management, and carefully timing stress-inducing procedures during this time may help reduce mortality.

## Figures and Tables

**Table 1:** The preliminary number of tested variables in the 13 thematic blocks together with the number of variables included into the multiblock partial least squares analysis.

Block Name	Variables in the Block (n)	Preselected Variables ^1^ (n)	
		MR21	MR90
Herd characteristics	7	4	3
Calving management	11	4	8
Colostrum management	13	7	3
Calf housing during the first four days of life	10	2	4
Calf feeding up to 21 days of age (post-colostrum)	11	2	3
Calf housing during 5–21 days of age	13	2	9
Calf feeding during 22–90 days of age	10	NA ^2^	3
Calf housing during 22–90 days of age	13	NA ^2^	8
Routine stress-inducing activities	4	3	0
General disease prevention measures	15	2	5
Pathogen-specific disease prevention measures	17	4	9
Disease status	10	1	2
External biosecurity	13	2	6
TOTAL	147	33	62

^1^ Selected using a negative binomial regression analysis (controlling for herd size) at a *p*-value limit ≤0.2, followed by a collinearity assessment. ^2^ NA—questions of the block were not included into younger age group univariable analyses due to unequivocal lack of possible association with mortality risk during the first 21 days of age.

## Data Availability

Data used in the present study was obtained from Estonian national registries of Estonian Agricultural Registers and Information Board and Estonian Livestock Performance Recording Ltd. under a confidentiality agreement and is not allowed to be made publicly accessible.

## Supplementary Materials

The following supporting information can be downloaded at https://www.mdpi.com/article/10.3390/ani15192780/s1. Supplementary Table S1. Diagnostic tests used for detecting disease-specific antibodies in heifer serum and bulk tank milk samples, and pathogen antigens in calf fecal samples; Supplementary Table S2. Descriptive statistics of categorical predictor variables and unconditional associations with herd annual calf mortality risk up to 21 days and 22–90 days of age in 118 Estonian dairy herds according to negative binomial regression analysis; Supplementary Table S3. Descriptive statistics of continuous predictor variables and unconditional associations with herd annual calf mortality risk up to 21 days and 22–90 days of age in 118 Estonian dairy herds according to negative binomial regression analysis; Supplementary Table S4. Variable importance indexes of the predictor variables in explaining composite outcome Y including herd annual calf mortality risk during the first 21 days and 22–90 days of age, as well as associations with the two fundamental variables of the Y, according to multiblock partial least squares analysis. References [58,107,108,109,110] are cited in Supplementary Materials.

## Abbreviations

The following abbreviations are used in this manuscript:

BHV-1	bovine herpesvirus 1
BlockImp	block importance index
BRSV	bovine respiratory syncytial virus
BTM	bulk tank milk
BVDV	bovine viral diarrhea virus
IgG	immunoglobulin G
IQR	interquartile range
mbPLS	multiblock partial least squares regression
MR21	mortality risk (%) during the first 21 days of age
MR90	mortality risk (%) during 22–90 days of age
OAG	older age group, calves aged 22–90 days
VarImp	variable importance index
YAG	younger age group, calves up to 21 days
