# Supplementary material for: Multiblock Analysis of Risk Factors and Management Areas of Calf Mortality in Large-Scale Dairy Herds
Source: Animals (Basel). 2025 Sep 24;15(19):2780. doi: 10.3390/ani15192780 (PMC12524161; doi:10.3390/ani15192780)
Supplement: Supplementary file 1 [file animals-15-02780-s001.zip › animals-3841179 Supplementary Table S1.pdf]

Supplementary Table S1. Diagnostic tests used for detecting disease-specific antibodies in heifer serum and bulk tank milk samples, and pathogen antigens in calf faecal samples

Pathogen	Diagnostic test	Diagnostic Se; Sp (%) in serum samples	Reference
Bovine Herpesvirus 1	IDEXX IBR gB X3 (IDEXX Laboratories, Inc.)<br>IDEXX IBR gE (IDEXX Laboratories, Inc.)	96.0; 99.0<br>72.0; 92.0	Kramps et al., 2004<br>Kramps et al., 2004
Bovine Viral Diarrhoea Virus	IDEXX BVDV Total Ab (IDEXX Laboratories, Inc.)	100.0; 95.0	Hanon et al., 2017
Bovine Respiratory Syncytial Virus	SVANOVIR® BRSV-Ab (Boehringer Ingelheim Svanova)	94.0; 100.0	Manufacturer (https://www.svanova.com/content/dam/internet/ah/svanova/dk_EN/documents/bovine/BRSV_Infosheet_b_V2.pdf)
Mycoplasma bovis	Monoscreen Ab ELISA (Bio-X Diagnostics S.A.)	49.1; 89.6	Andersson et al., 2019
Mycobacterium avium spp. *paratuberculosis*	*Mycobacterium paratuberculosis* Test Kit for Cattle PARACHEK® 2 (Prionics AG)	71.8; 100.0	Manufacturer (http://tools.thermofisher.com/content/sfs/brochures/animalhealth_flyer_parachek2_map_CO121142.pdf)
Salmonella Dublin	PrioCHECK® Salmonella Ab bovine Dublin (Prionics AG)	85.0; 85.0	Nielsen and Ersbøll, 2004
Bovine rotavirus, coronavirus, *E. coli* F5, *Cryptosporidium parvum*	BIO K 348 Multiscreen AgELISA Calf digestive (Bio-X Diagnostics)	100.0; 98.0 (rotavirus)<br>90.0; 95.0 (coronavirus)<br>100.0; 90.9 (*E. coli* F5)<br>90.9; 97.1 (*C. parvum*)	Manufacturer (https://www.biox.com/en/bio-k-348-multiscreen-agelisa-calf-digestive-sandwich-double-wells-p-309/)

[107] Andersson, A.M., Aspán, A., Wisselink, H.J., Smid, B., Ridley, A., Pelkonen, S., Autio, T., Lauritsen, K.T., Kensø, J., Gaurivaud, P., Tardy, F., 2019. A European inter-laboratory trial to evaluate the performance of three serological methods for diagnosis of Mycoplasma bovis infection in cattle using latent class analysis. BMC Vet. Res. 15. https://doi.org/10.1186/s12917-019-2117-0

[108] Hanon, J.B., De Baere, M., De la Ferté, C., Roelandt, S., Van der Stede, Y., Cay, B., 2017. Evaluation of 16 commercial antibody ELISAs for the detection of bovine viral diarrhea virus–specific antibodies in serum and milk using well-characterized sample panels. J. Vet. Diagnostic Investig. 29, 833–843. https://doi.org/10.1177/1040638717724839

[109] Kramps, J.A., Banks, M., Beer, M., Kerkhofs, P., Perrin, M., Wellenberg, G.J., Oirschot, J.T.V., 2004. Evaluation of tests for antibodies against bovine herpesvirus 1 performed in national reference laboratories in Europe. Vet. Microbiol. 102, 169–181. https://doi.org/10.1016/j.vetmic.2004.07.003

[110] Nielsen, L.R., Ersbøll, A.K., 2004. Age-stratified validation of an indirect Salmonella Dublin serum enzyme-linked immunosorbent assay for individual diagnosis in cattle. J. Vet. Diagnostic Investig. 16, 212–218. https://doi.org/10.1177/104063870401600306
