# Supplementary material for: Multiblock Analysis of Risk Factors and Management Areas of Calf Mortality in Large-Scale Dairy Herds
Source: Animals (Basel). 2025 Sep 24;15(19):2780. doi: 10.3390/ani15192780 (PMC12524161; doi:10.3390/ani15192780)
Supplement: Supplementary file 1 [file animals-15-02780-s001.zip › animals-3841179 Supplementary Table S2.pdf]

Supplementary Table S2. Descriptive statistics of categorical predictor variables and unconditional associations with herd annual calf mortality risk up to 21 days and 22-90 days of age in 118 Estonian dairy herds according to negative binomial regression analysis

Block / Variable	Categories	n	Mortality risk of up to 21 day old calves (SD)	P-value[a]	Mortality risk of 22-90 day old calves (SD)	P-value[a]
Herd characteristics						
Region[b]	Northeast	46	6.6 (5.2)	0.284	2.8 (1.8)	0.735
	Southeast	24	5.8 (6.6)		2.4 (2.0)	
	Southwest	30	5.9 (5.1)		2.8 (2.0)	
	Northwest	18	4.1 (4.0)*		3.0 (2.9)	
Herd number of cows	<250 cows	30	5.5 (4.6)	0.358	2.9 (2.8)	0.584
	250−459 cows	29	6.2 (5.6)		2.7 (1.5)	
	460−629 cows	29	4.8 (5.2)		2.3 (2.0)	
	≥630 cows	30	7.0 (5.9)		3.0 (1.7)	
Herd predominant breed	Estonian Holstein Friesian breed ≥75%	96	5.4 (4.6)	0.145	2.6 (2.0)	0.111
	Estonian Red breed ≥75%	9	7.9 (7.0)		4.1 (2.7)*	
	Mix of breeds	13	7.9 (8.2)		2.4 (1.5)	
Perspective for changes in herd size	Increase	64	6.6 (5.5)	0.094	3.0 (2.1)	0.110
	Not to change	54	5.1 (5.0)		2.4 (2.0)	
Time since building/reconstructing the calf barn	<10 years	44	6.4 (6.2)	0.021	2.8 (2.0)	0.658
	10−20 years	37	4.2 (4.1)*		2.5 (1.8)	
	>20 years	37	7.0 (5.0)		2.8 (2.4)	
Milking system	Carousel milking parlour	6	8.5 (9.3)	0.619	4.0 (1.6)	0.329
	Parallel milking parlour	53	6.0 (5.1)		2.6 (1.8)	
	Herringbone milking parlour	16	6.3 (5.6)		2.4 (1.8)	

	Robot milking	34	5.3 (4.8)		2.9 (2.7)	
	Combined or other[c]	9	6.7 (5.9)		2.0 (1.2)	
Calving management						
Main calving system during the last year	Tied	16	6.6 (5.1)	0.664	3.5 (2.5)	0.331
	Individual pen	21	4.9 (4.5)		2.4 (1.3)*	
	Group pen	78	6.0 (5.6)		2.7 (2.1)	
	Combined	3	6.2 (7.0)		2.0 (0.8)*	
Number of animals in the calving pen	One	38	5.8 (4.9)	0.986	2.9 (2.0)	0.735
	Two to seven	38	6.0 (5.7)		2.5 (2.0)	
	≥Eight	42	5.8 (5.5)		2.8 (2.2)	
Using bedding in calving pen	No	3	5.6 (4.3)	0.939	5.6 (6.3)	0.026
	Yes	115	5.9 (5.4)		2.6 (1.9)*	
Calving pen bedding material	Straw or hay	82	5.9 (5.7)	0.979	2.6 (1.6)	0.122
	Sawdust	15	6.3 (4.2)		2.8 (2.1)	
	Combined or other	18	5.6 (4.7)		3.0 (2.7)	
	None	3	5.6 (4.3)		5.6 (6.3)*	
Time before the expected calving when a cow/heifer is placed to a calving pen	Only for foetus ejection	31	5.4 (4.5)	0.191	2.7 (1.4)	0.231
	A few days before calving	15	4.3 (5.0)		1.9 (1.0)	
	More than a few days before calving	72	6.4 (5.7)		2.9 (2.4)	
Time after calving when a cow/heifer is kept in the calving pen	One hour or less	32	5.9 (5.0)	0.863	2.1 (1.5)	0.033
	Two to six hours	26	5.9 (6.3)		2.5 (1.5)	
	Seven to 48 hours	31	5.4 (5.7)		2.9 (2.2)*	
	Over 48 hours	29	6.5 (4.7)		3.4 (2.7)**	
24-hour calving monitoring	No	17	5.3 (4.0)	0.581	2.8 (3.0)	0.995

	Yes	101	6.0 (5.5)		2.7 (1.9)	
Use of technical tools to monitor calvings	No	93	6.3 (5.7)	0.046	2.9 (2.2)	0.081
	Yes	25	4.3 (3.6)*		2.1 (1.6)	
Proportion of animals with dirty lower hind limb (score 3 and 4) in the calving group[h]	<10%	49	4.8 (4.7)	0.075	2.4 (2.0)	0.180
	10−39%	35	6.2 (5.5)		2.8 (1.9)	
	≥40%	34	7.2 (5.9)*		3.1 (2.4)	
Proportion of animals with dirty udder (score 3 and 4) in the calving group[h]	<10%	76	5.5 (5.6)	0.261	2.3 (1.7)	0.001
	≥10%	42	6.6 (4.8)		3.5 (2.4)***	
Proportion of animals with dirty upper hind limb and flank (score 3 and 4) in the calving group[h]	<10%	30	4.5 (5.6)	0.061	2.2 (1.9)	0.169
	10−24%	27	6.2 (4.1)		3.2 (2.2)	
	25−49%	28	5.0 (4.5)		2.4 (2.5)	
	≥50%	33	7.6 (6.3)**		3.0 (1.6)	
Colostrum management						
Milking the majority (>90%) of cows during the first two hours after calving	No	53	6.3 (5.8)	0.187	2.8 (2.4)	0.255
	Yes	60	5.2 (4.7)		2.5 (1.6)	
	The calf suckles by itself	5	9.7 (6.4)		4.0 (3.3)	
Time after birth when first colostrum is fed	≤1 hour	54	5.0 (4.8)	0.133	2.8 (1.7)	0.748
	1,5−2 hours	47	6.9 (6.3)*		2.6 (2.0)	
	≥3 hours	17	5.8 (3.8)		2.8 (3.1)	
The amount of colostrum fed at first feeding	≤2 litres	46	6.6 (5.8)	0.131	3.1 (2.5)	0.131
	>2 litres	66	5.1 (4.9)		2.4 (1.6)	
	Ad libitum	6	8.7 (6.2)		3.6 (3.2)	
Type of colostrum fed to calves	Fresh colostrum	45	5.7 (4.8)	0.812	2.8 (2.5)	0.404

	Frozen colostrum	17	6.7 (6.4)		3.1 (1.6)	
	Fresh and frozen colostrum	33	6.1 (5.8)		2.8 (1.9)	
	Cooled colostrum and combinations with it or all types used simultaneously	23	5.3 (5.1)		2.1 (1.6)	
Equipment used for first colostrum feeding	Nipple bucket	23	4.8 (5.5)	0.551	2.5 (2.7)	0.673
	Nipple bottle	74	6.0 (5.3)		2.7 (1.9)	
	Oesophageal tube	11	6.0 (6.0)		1.7 (1.5)	
	Bucket	1	4.0 (NA)		0.8 (NA)	
	The calf suckles by itself (always or occasionally)	9	8.2 (5.4)		3.4 (2.7)	
Measuring the quality of first colostrum in majority (>90%) of the cases	No[d]	60	6.6 (5.6)	0.057	3.2 (2.5)	0.001
	Yes	58	5.1 (5.0)		2.2 (1.4)**	
Measuring the temperature of the colostrum before feeding	No	44	6.5 (5.3)	0.299	3.0 (2.3)	0.274
	Yes[d]	74	5.5 (5.4)		2.6 (1.9)	
Feeding pooled colostrum	No	86	6.1 (5.6)	0.364	2.6 (2.1)	0.420
	Yes	32	5.3 (4.7)		2.9 (2.0)	
Always feeding first colostrum with oesophageal tube if the calf does not drink by itself	No	64	6.5 (5.5)	0.097	3.0 (2.4)	0.108
	Yes	54	5.1 (5.1)		2.4 (1.6)	
Always recording information about first feeding	No	41	6.3 (5.8)	0.517	3.0 (2.7)	0.358
	Yes	77	5.7 (5.1)		2.6 (1.7)	
Testing passive (colostral) immunity of calves up to seven days of age	No	78	5.8 (5.5)	0.866	2.6 (2.2)	0.481
	Yes	40	6.0 (5.1)		2.9 (1.9)	
Calf housing during the first four days of life						

Keeping system during this period	Individual pen	112	5.8 (5.4)	0.598	2.7 (2.0)	0.215
	Individual and group pen[e]	6	7.0 (5.3)		3.8 (2.6)	
Calf pen floor material	Wood (solid or slatted)	56	5.9 (5.3)	0.996	2.7 (2.0)	0.318
	Concrete	37	5.8 (5.0)		2.4 (1.9)	
	Mat or mattress	9	6.2 (4.4)		3.8 (2.6)	
	Combined or other	16	5.9 (7.1)		2.9 (2.2)	
Calf pen wall material	Laminated plywood	68	5.5 (5.1)	0.242	2.6 (2.0)	0.767
	Plastic	20	6.4 (7.3)		2.8 (2.3)	
	Metal	10	4.0 (2.5)		2.4 (1.6)	
	Combined or other	20	7.5 (5.0)		3.1 (2.4)	
Other animal groups in the same (air)room	None	6	4.7 (2.7)	0.567	4.1 (2.8)	0.167
	Youngstock (calves and heifers)	17	7.0 (7.5)		2.4 (1.6)	
	Youngstock and adult cows (including sick cows)	95	5.8 (5.0)		2.7 (2.1)	
Calves being kept in the same room with cows	No	26	6.4 (6.6)	0.815	2.7 (2.0)	0.779
	Yes, distance to cows up to 3m	60	5.6 (5.1)		2.6 (2.1)	
	Yes, distance to cows over 3m	32	5.9 (4.8)		2.9 (2.0)	
Always washing calf pen before a new calf is placed into the pen	No	30	7.4 (5.1)	0.066	3.4 (2.3)	0.028
	Yes	88	5.4 (5.4)		2.5 (2.0)*	
Always having new clean bedding when a new calf is placed into the pen	No	8	7.2 (5.4)	0.487	3.7 (2.4)	0.166
	Yes	110	5.8 (5.4)		2.7 (2.0)	
Possibility of one calf defecating into another calf's pen	No	76	5.6 (5.8)	0.431	2.6 (1.8)	0.305
	Yes	42	6.4 (4.4)		3.0 (2.5)	
Direct contact between calves possible	No	22	6.5 (5.9)	0.538	2.8 (1.9)	0.859
	Yes	96	5.7 (5.2)		2.7 (2.1)	

Staff routinely entering the pen to perform daily procedures	No	74	5.9 (5.5)	0.952	2.6 (2.1)	0.328
	Yes	44	5.9 (5.1)		2.9 (2.0)	
Calf feeding up to 21 days of age (post-colostrum)						
Number of daily milk feedings	Two	77	6.1 (5.3)	0.606	2.7 (2.1)	0.117
	Three[f]	20	6.2 (6.3)		2.2 (0.8)	
	Automatic milk feeder[f]	21	4.9 (4.8)		3.4 (2.7)	
Equipment used for milk feeding	Individual nipple bucket	57	5.3 (4.9)	0.213	2.4 (1.8)	0.167
	Bucket	24	7.5 (6.0)		3.0 (2.0)	
	Automatic milk feeder[f]	21	4.9 (4.8)		3.4 (2.7)*	
	Combined or other	16	6.9 (6.3)		2.5 (2.0)	
Type of milk feed used	Milk replacer	52	6.0 (5.4)	0.846	2.6 (2.0)	0.776
	Unpasteurized whole milk	33	6.1 (6.1)		2.8 (2.0)	
	Combined or other	33	5.5 (4.6)		2.9 (2.3)	
Feeding calves milk collected from cows undergoing antibiotic treatment or in a withdrawal period	No[g]	87	5.8 (5.3)	0.663	2.5 (2.0)	0.057
	Yes[e]	31	6.2 (5.5)		3.3 (2.2)	
Feeding calves unpasteurized waste milk (including milk with high SCC)	No[g]	76	5.9 (5.5)	0.952	2.6 (2.0)	0.436
	Yes[e]	42	5.9 (5.2)		2.9 (2.1)	
Crude fibre content of the milk replacer used currently	≤0.1%[e]	84	5.6 (4.5)	0.434	2.7 (2.1)	0.634
	>0.1%[e]	7	3.0 (2.1)		2.2 (1.6)	
	Milk replacer is not used	27	6.4 (6.6)		3.0 (2.1)	
Starter feed/muesli freely available	No	6	5.8 (4.9)	0.969	2.9 (2.1)	0.876
	Yes	112	5.9 (5.4)		2.7 (2.1)	

Hay/straw freely available	No	35	5.7 (4.4)	0.781	2.4 (1.7)	0.308
	Yes	83	6.0 (5.7)		2.8 (2.2)	
Silage freely available	No	112	6.1 (5.4)	0.042	2.7 (2.1)	0.917
	Yes	6	2.7 (1.9)*		2.8 (1.6)	
Calf housing during 5−21 days of age						
Prevailing keeping system and maximum group size	Individual pen	61	5.4 (5.1)	0.581	2.5 (1.9)	0.140
	Group pen, 2−12 animals per pen[e]	35	6.2 (5.0)		2.7 (1.9)	
	Group pen, ≥13 animals per pen[e]	22	6.6 (6.6)		3.4 (2.7)*	
Individual box or group pen floor material	Wood (solid or slatted)	28	6.7 (5.5)	0.568	2.4 (1.6)	0.128
	Concrete	54	5.7 (5.2)		2.6 (2.3)	
	Mat or mattress	4	3.5 (1.8)		5.0 (3.6)*	
	Combined or other	32	5.8 (5.9)		2.9 (1.8)	
Individual box or group pen wall material	Laminated plywood	34	6.0 (5.0)	0.917	2.4 (1.4)	0.597
	Plastic	19	5.2 (6.1)		2.5 (2.4)	
	Metal	16	5.8 (4.7)		3.1 (3.0)	
	Combined or other	49	6.1 (5.6)		2.9 (2.0)	
Other animal groups in the same (air)room	No other animal groups	5	2.9 (2.6)	0.228	3.7 (3.4)	0.477
	Youngstock (calves and heifers)	36	6.1 (5.7)		2.5 (2.3)	
	Youngstock and adult cows (including sick cows)	77	6.0 (5.3)		2.8 (1.9)	
Calves being kept in the same room with cows	No	43	5.8 (5.6)	0.939	2.6 (2.4)	0.504
	Yes, distance to cows up to 3 m	49	6.1 (5.4)		2.6 (1.7)	
	Yes, distance to cows over 3 m	26	5.7 (5.0)		3.1 (2.1)	
	No or sometimes	31	6.9 (5.2)	0.324	3.8 (2.7)	0.002

Always washing calf box before a new calf is placed into the box	Yes	74	5.7 (5.5)		2.3 (1.6)***	
	No calf movements during this period	13	4.7 (4.8)		2.5 (2.0)	
Always having new clean bedding when a new calf is placed into the box	No or sometimes	13	6.2 (4.9)	0.634	3.8 (3.3)	0.131
	Yes	92	6.0 (5.5)		2.6 (1.8)*	
	No calf movements during this period	13	4.7 (4.8)		2.5 (2.0)	
Possibility of one calf defecating into another calf's box or another pen	No	55	5.9 (6.2)	0.924	2.5 (1.9)	0.173
	Yes[e]	63	5.8 (4.5)		3.0 (2.2)	
Direct contact between calves possible	No	15	5.4 (4.9)	0.662	3.2 (2.4)	0.345
	Yes[e]	103	6.0 (5.4)		2.7 (2.0)	
Forced air ventilation in calves' room	No[e]	85	6.0 (5.0)	0.578	3.0 (2.3)	0.005
	Yes	33	5.5 (6.2)		1.9 (1.1)**	
Access to outdoor area	No	112	6.1 (5.4)	0.054	2.6 (1.9)	0.019
	Yes[e]	6	2.8 (3.3)*		4.7 (4.2)*	
Staff routinely entering the pen to perform daily procedures	No	66	6.1 (5.7)	0.549	2.4 (1.8)	0.066
	Yes[e]	52	5.6 (4.9)		3.1 (2.4)	
Calf feeding during 22−90 days of age						
Milk feeding system	Individual feeding two or three times per day	46	NA		2.5 (2.0)	0.115
	Automatic milk feeder	65	NA		2.7 (1.7)	
	Combination of individual feeding and automatic milk feeder	7	NA		4.3 (4.4)*	
Equipment used for milk feeding	Individual nipple bucket	5	NA		1.5 (0.7)	0.469
	Bucket	15	NA		2.5 (2.0)	

	Automatic milk feeder[f]	73	NA	2.8 (2.1)	
	Combined or other	25	NA	2.8 (2.2)	
Type of milk feed	Milk replacer	86	NA	2.6 (1.8)	0.311
	Unpasteurized whole milk	14	NA	3.5 (2.3)	
	Combined or other	18	NA	2.7 (2.9)	
Feeding waste milk from cows receiving antibiotic treatment or in a withdrawal period	No[g]	101	NA	2.6 (1.9)	0.262
	Yes[e]	17	NA	3.2 (2.9)	
Feeding calves unpasteurized waste milk (including milk with high SCC)	No[g]	95	NA	2.7 (1.9)	0.984
	Yes[e]	23	NA	2.7 (2.7)	
Crude fibre content of the milk replacer used currently	≤0.1%[e]	94	NA	2.7 (2.1)	0.969
	>0.1%[e]	14	NA	2.6 (2.4)	
	Milk replacer is not used	10	NA	2.8 (1.7)	
Starter feed/muesli freely available	No	2	NA	4.6 (1.1)	0.236
	Yes	116	NA	2.7 (2.1)	
Hay/straw freely available	No	4	NA	2.6 (1.6)	0.967
	Yes	114	NA	2.7 (2.1)	
Silage freely available	No	59	NA	3.1 (2.3)	0.047
	Yes	59	NA	2.4 (1.7)*	
Calf housing during 22−90 days of age					
Keeping system during this period	Individual pen	5	NA	2.3 (2.5)	0.569
	Group pen, ≥1.5 m^2 per calf	100	NA	2.7 (2.1)	

	Group pen, <1.5 m^2 per calf	5	NA	3.9 (2.1)	
	Individual and group pen[e]	8	NA	2.4 (2.0)	
Maximum number of animals in one group pen	No group pens	5	NA	2.3 (2.5)	0.114
	≤15 animals in one group pen	61	NA	2.4 (1.8)	
	≥16 animals in one group pen	52	NA	3.1 (2.3)	
Other animal groups in the same (air)room	No other animal groups	15	NA	3.4 (2.4)	0.320
	Youngstock (calves and heifers)	47	NA	2.5 (2.3)	
	Youngstock and adult cows (including sick cows)	56	NA	2.8 (1.7)	
Calves being kept in the same room with cows	No	63	NA	2.7 (2.3)	0.626
	Yes, distance to cows up to 3 m	33	NA	2.5 (1.7)	
	Yes, distance to cows over 3 m	22	NA	3.1 (1.8)	
Principle of adding calves to group pens	As a group	49	NA	2.4 (1.7)	0.199
	Continuously	64	NA	3.0 (2.3)	
	No group pens	5	NA	2.3 (2.5)	
Maximum age difference of calves in the same group pen	2−9 days	25	NA	1.9 (1.2)	0.050
	10−19 days	30	NA	2.7 (1.9)*	
	20−39 days	29	NA	3.0 (2.1)**	
	≥40 days	29	NA	3.2 (2.6)**	
	No group pens	5	NA	2.3 (2.5)	
Proportion of calves with a cleanliness score of 3 and 4 of the lower hind limbs[h]	<10%	97	NA	2.7 (2.1)	0.801
	≥10%	21	NA	2.8 (1.8)	
Proportion of calves with a cleanliness score of 3 and 4 of the upper hind limb and flank[h]	<10%	73	NA	2.9 (2.3)	0.141
	≥10%	45	NA	2.4 (1.5)	

Continuous faeces movement between different calf groups	No (including farms with no group pens)	76	NA		2.6 (2.1)	0.328
	Yes	42	NA		3.0 (2.0)	
Direct contact between calves possible	No	23	NA		2.2 (1.8)	0.183
	Yes[e]	95	NA		2.8 (2.1)	
Forced air ventilation in calves' room	No[e]	85	NA		2.9 (2.2)	0.128
	Yes (including farms with no indoor keeping)	33	NA		2.3 (1.6)	
Access to outdoor area	No	99	NA		2.6 (1.9)	0.121
	Yes	19	NA		3.4 (2.8)	
Routine stress-inducing activities						
Transporting calves aged up to 21 days	No	101	6.1 (5.6)	0.239	2.7 (2.1)	0.856
	Yes	17	4.7 (3.7)		2.6 (2.0)	
Transporting calves aged between 22 and 90 days	No	98	NA		2.8 (2.1)	0.346
	Yes	20	NA		2.3 (1.6)	
Disbudding calves	Only heifer calves	108	5.6 (5.3)	0.059	2.7 (2.1)	0.633
	Heifer and bull calves	10	9.2 (5.4)		3.0 (1.7)	
Maximum age of calves at disbudding	≤ 20 days	28	4.7 (5.3)	0.057	2.7 (1.8)	0.595
	21−29 days	43	7.3 (6.1)*		2.9 (2.5)	
	≥30 days	47	5.3 (4.3)		2.5 (1.8)	
General disease prevention measures						
Employing wet disinfection in calving pen	No	108	6.1 (5.5)	0.022	2.7 (2.0)	0.845
	Yes	10	3.2 (2.3)*		2.6 (2.8)	

Use of calving pens for sick cows, including cows planned to cull	No	53	5.7 (4.8)	0.718	2.2 (1.4)	0.007
	Yes	65	6.0 (5.8)		3.2 (2.4)**	
Housing colostrum period cows together with sick cows	No	30	6.1 (5.4)	0.759	2.5 (1.5)	0.414
	Yes	88	5.8 (5.4)		2.8 (2.2)	
Pasteurization of colostrum in majority (>90%) of the cases	No	114	5.9 (5.4)	0.490	2.7 (2.1)	0.965
	Yes	4	4.7 (1.9)		2.7 (1.5)	
Disinfecting the umbilical cord in majority (>90%) of the newborn calves	No	25	5.7 (4.9)	0.819	3.0 (2.7)	0.522
	Yes	93	5.9 (5.5)		2.7 (1.9)	
Drying (including heating lamps) the majority (>90%) of newborn calves	No	51	5.7 (5.5)	0.654	2.4 (2.3)	0.154
	Yes, seasonally	17	7.0 (6.4)		3.3 (1.8)	
	Yes, always	50	5.7 (4.9)		2.9 (1.8)	
Always applying a wet disinfectant before placing a newborn calf to the pen	No	53	5.8 (4.6)	0.894	3.1 (2.5)	0.069
	Yes	65	6.0 (5.9)		2.4 (1.6)	
Always applying a wet disinfectant before introducing a new calf to the pen during 5-21 days of age	No or sometimes	51	6.6 (5.6)	0.318	3.3 (2.5)	0.009
	Yes	54	5.5 (5.2)		2.2 (1.4)**	
	No calf movements during this period	13	4.7 (4.8)		2.5 (2.0)	
Always applying a wet disinfectant before introducing a new calf to the pen during 22-90 days of age	No	102	NA		2.8 (2.1)	0.472
	Yes	16	NA		2.4 (2.2)	
Cleaning teats of the nipple bucket after every calf during the first 21 days of calf's life	No or sometimes	47	5.2 (5.1)	0.276	2.5 (1.6)	0.571
	Yes	34	5.6 (4.9)		2.8 (2.2)	
	Nipple buckets are not used	37	7.0 (6.0)		2.9 (2.5)	

Cleaning teats of the nipple bucket after every calf during 22-90 days of calf's life	No	6	NA		2.1 (1.5)	0.367
	Yes	11	NA		3.4 (2.9)	
	Nipple buckets are not used	101	NA		1.7 (2.0)	
Separating sick calves during group housing period	No or sometimes	86	5.8 (5.0)	0.644	2.6 (2.1)	0.492
	Yes or no group pens	32	6.2 (6.3)		2.9 (2.1)	
Summary report of last year´s calf health data has been made	No	78	6.1 (5.7)	0.487	2.8 (2.2)	0.750
	Yes	40	5.5 (4.6)		2.6 (1.8)	
Prophylactic/metaphylactic administration of injectable antibiotics for calves during the last year	No	84	6.1 (5.7)	0.193	2.9 (2.2)	0.094
	Yes, during some period	11	7.1 (4.3)		2.7 (2.0)	
	Yes, continuously	23	4.4 (4.3)		2.0 (1.4)*	
Routine usage of oral antibiotics for calves during the last year	No	102	6.0 (5.5)	0.379	2.8 (2.1)	0.645
	Yes	16	4.9 (4.6)		2.5 (1.6)	
Pathogen-specific disease prevention measures						
Routine usage of oral drugs against cryptosporidiosis for calves during the last year	No	88	5.4 (5.1)	0.078	2.7 (2.2)	0.606
	Yes	30	7.3 (5.9)		2.9 (1.6)	
Routine usage of oral drugs against eimeriosis for calves during the last year	No	98	5.7 (5.3)	0.377	2.7 (2.2)	0.954
	Yes	20	6.8 (5.7)		2.7 (1.5)	
Vaccinating calves against bovine respiratory syncytial virus during the last year	No	98	6.1 (5.5)	0.400	2.7 (2.1)	0.580
	Yes	20	5.1 (4.5)		2.9 (2.2)	
	No	110	6.0 (5.4)	0.433	2.8 (2.1)	0.061

Vaccinating calves against bovine herpesvirus 1 during the last year	Yes	8	4.6 (3.8)		1.5 (1.1)	
Vaccinating calves against bovine viral diarrhea virus during the last year	No	110	6.0 (5.4)	0.433	2.8 (2.1)	0.061
	Yes	8	4.6 (3.8)		1.5 (1.1)	
Vaccinating calves against bovine parainfluenza virus 3 during the last year	No	100	6.1 (5.5)	0.379	2.7 (2.1)	0.859
	Yes	18	5.0 (4.4)		2.8 (2.0)	
Vaccinating calves against *Mannheimia haemolytica* during the last year	No	111	5.9 (5.4)	0.700	2.8 (2.1)	0.079
	Yes	7	5.2 (3.7)		1.5 (1.2)	
Vaccinating calves against ringworm during the last year	No	67	6.2 (6.1)	0.443	2.7 (2.1)	0.681
	Yes	51	5.5 (4.3)		2.8 (2.0)	
Vaccinating cows and heifers against bovine respiratory syncytial virus during the last 3-5 years	No	108	5.9 (5.5)	0.925	2.8 (2.1)	0.233
	Yes	10	5.8 (2.7)		2.0 (2.2)	
Vaccinating cows and heifers against bovine herpesvirus 1 during the last 3-5 years	No	101	6.3 (5.6)	0.015	2.8 (2.1)	0.233
	Yes	17	3.6 (2.2)*		2.2 (2.1)	
Vaccinating cows and heifers against bovine viral diarrhea virus during the last 3-5 years	No	108	5.7 (5.3)	0.281	2.7 (2.1)	0.717
	Yes	10	7.7 (6.0)		2.5 (2.4)	
Vaccinating cows and heifers against bovine parainfluenza virus 3 during the last 3-5 years	No	110	5.9 (5.5)	0.985	2.8 (2.1)	0.212
	Yes	8	5.9 (2.9)		1.9 (2.3)	
	No	110	5.9 (5.5)	0.985	2.8 (2.1)	0.212

Vaccinating cows and heifers against *Mannheimia haemolytica* during the last 3-5 years	Yes	8	5.9 (2.9)		1.9 (2.3)	
Vaccinating cows and heifers against ringworm during the last 3-5 years	No	104	6.0 (5.5)	0.702	2.7 (2.1)	0.821
	Yes	14	5.4 (4.4)		2.8 (1.8)	
Vaccinating cows and heifers against mastitis pathogens during the last 3-5 years	No	114	5.8 (5.3)	0.316	2.7 (2.1)	0.444
	Yes	4	8.8 (8.0)		2.0 (1.0)	
Vaccinating cows and heifers before parturition against rotavirus, coronavirus and enterotoxignic *E. coli* during the last 3-5 years	No	100	5.6 (4.8)	0.179	2.6 (2.1)	0.217
	Yes	18	7.5 (7.9)		3.2 (1.8)	
Vaccinating cows and heifers against clostridiosis during the last 3-5 years	No	117	5.9 (5.4)	0.969	2.7 (2.1)	0.667
	Yes	1	5.7 (NA)		1.9 (NA)	
Disease status						
Herd bovine respiratory syncytial virus status	Negative	5	4.3 (4.0)	0.091	2.5 (1.9)	0.974
	BTM positive, heifers negative	26	4.6 (4.2)		2.7 (2.6)	
	BTM and heifers positive	63	6.9 (6.1)		2.8 (1.9)	
	Vaccinating herds	24	4.8 (4.1)		2.6 (2.0)	
Herd *Mycobacterium avium* spp. *paratuberculosis* status	Negative	115	5.8 (5.3)	0.621	2.7 (2.1)	0.779
	BTM or heifers positive	3	7.4 (6.7)		2.4 (1.0)	
Herd bovine herpesvirus 1 status	Negative	26	6.1 (5.4)	0.457	2.7 (2.6)	0.728
	BTM positive, heifers negative	33	6.6 (5.6)		3.0 (2.3)	
	BTM and heifers positive	29	6.1 (4.8)		2.6 (1.4)	
	Vaccinating herds	30	4.7 (5.7)		2.4 (1.9)	

Herd bovine viral diarrhea virus status	Negative	80	5.9 (5.4)	0.817	2.7 (2.2)	0.984
	BTM and/or heifers positive	29	5.6 (4.3)		2.7 (1.7)	
	Vaccinating herds	9	6.8 (8.0)		2.6 (2.2)	
Herd *Mycoplasma bovis* status	Negative	60	6.0 (5.9)	0.858	2.8 (2.4)	0.901
	BTM and/or heifers positive	58	5.8 (4.8)		2.7 (1.7)	
Herd *Salmonella* Dublin status	Negative	90	5.6 (5.3)	0.435	2.7 (2.2)	0.735
	BTM positive, heifers negative	15	6.0 (4.5)		2.9 (1.5)	
	BTM positive or negative, heifers positive	13	7.6 (6.3)		3.0 (1.7)	
Calf faecal samples testing positive for bovine rotavirus	No	45	6.1 (6.0)	0.643	3.2 (2.5)	0.036
	Yes	72	5.7 (4.9)		2.4 (1.7)*	
Calf faecal samples testing positive for bovine coronavirus	No	106	5.8 (5.4)	0.771	2.7 (2.1)	0.389
	Yes	11	6.3 (5.5)		3.2 (1.8)	
Calf faecal samples testing positive for enterotoxigenic *Escherichia coli*	No	101	5.8 (5.5)	0.791	2.7 (2.1)	0.701
	Yes	16	6.1 (4.5)		2.9 (2.1)	
Calf faecal samples testing positive for *Cryptosporidium parvum*	No	26	6.1 (5.6)	0.761	2.2 (1.7)	0.147
	Yes	91	5.8 (5.3)		2.9 (2.2)	
External biosecurity						
Continuous movement of cattle between different units of the same owner	No	93	6.1 (5.7)	0.349	2.8 (2.3)	0.265
	Yes	25	5.1 (4.0)		2.3 (1.1)	
Farm purchased and introduced new animals to the herd during the last three years	No	59	5.7 (4.9)	0.707	2.6 (1.6)	0.668
	Yes	59	6.1 (5.8)		2.8 (2.5)	

Grazing cows and/or heifers	No	48	6.2 (5.5)	0.280	2.6 (1.8)	0.001
	Only youngstock	29	4.5 (4.0)		2.1 (1.6)	
	Only cows (including dry cows)	15	7.0 (4.2)		4.6 (3.4)**	
	Dry cows and youngstock	26	6.3 (6.8)		2.5 (1.5)	
Pastures/outdoor areas border pastures of other cattle owners/other units of the same company	No	75	5.7 (5.2)	0.380	2.7 (2.1)	0.941
	Yes	12	4.7 (6.0)		2.7 (2.4)	
	No outdoor areas	31	6.8 (5.4)		2.8 (1.8)	
Animals participated in animal exhibition or auction during the last three years	No	96	5.9 (5.7)	0.964	2.9 (2.2)	0.110
	Yes	22	5.9 (3.7)		2.1 (1.3)	
Veterinarian/veterinary assistant/AI technician/farm employee visit other herds or other units of the same owner	No	16	6.2 (6.7)	0.792	2.7 (2.0)	0.949
	Yes	102	5.8 (5.1)		2.7 (2.1)	
Veterinarian/veterinary assistant/AI technician provide service to other herds	No	30	5.7 (5.4)	0.773	2.6 (1.9)	0.661
	Yes	88	6.0 (5.4)		2.8 (2.2)	
Farm workers change clothes on the farm	No or sometimes	11	7.0 (5.6)	0.463	3.9 (3.4)	0.048
	Yes	107	5.8 (5.3)		2.6 (1.9)*	
Visitors who come into contact with the animals wear protective clothing and footwear	No or sometimes	50	6.1 (5.9)	0.737	3.2 (2.1)	0.031
	Yes	68	5.8 (4.9)		2.4 (2.0)*	
Service providers disinfect their equipment before entering the farm	No or sometimes	21	4.6 (4.1)	0.167	2.8 (2.6)	0.888
	Yes (including farms who do not have service providers)	97	6.2 (5.6)		2.7 (2.0)	
	No	90	6.1 (5.8)	0.352	2.9 (2.2)	0.098

Operational disinfection mats/baths available at all times at people's entry points	Yes	28	5.2 (3.7)		2.2 (1.4)	
Hand disinfectants at people's entry points	No	104	6.1 (5.6)	0.285	2.8 (2.2)	0.164
	Yes	14	4.6 (3.2)		2.1 (1.1)	
Distance (m) between the farm and the loading place of the carcasses	≤ 5 m	32	7.4 (6.7)	0.016	2.7 (1.8)	0.674
	6−19 m	24	3.6 (2.7)**		2.8 (2.4)	
	20−49 m	21	5.6 (5.9)		2.3 (1.6)	
	≥50 m	41	6.2 (4.7)		2.9 (2.3)	

[a]Number of cows was included to the models as a fixed effect. *P ≤ 0.05, **P ≤ 0.01 and ***P ≤ 0.001 compared to the first reference category

[b]Northeast Estonia: Ida-Viru, Lääne-Viru, Jõgeva, Järva county; Southeast Estonia: Tartu, Valga, Võru, Põlva county; Southwest Estonia: Pärnu, Viljandi, Saare county; Northwest Estonia: Harju, Rapla, Lääne, Hiiu county

[c]Other (n = 8), robot carousel (n = 1)

[d]Including farms where the calf suckles by itself

[e]Including farms, where this practice was used only for some period of time or only for certain animal group

[f]Some or whole period

[g]Including farms where whole milk is not fed

[h]Cleanliness of the animals was scored on a four-level scale: 0 = completely clean, 1 = slightly dirty, 2 = moderately dirty, and 3 = very dirty, based on the scoring chart described by Cook and Reinemann (2007). If case of different cleanliness states in the right and left side or body parts, the dirtier side was chosen for the assessment

References:

[58] Cook, N., Reinemann, D., 2007. A tool box for assessing cow, udder and teat hygiene, in: Annual Meeting of the NMC. pp. 21–24.
