# Supplementary material for: Multiblock Analysis of Risk Factors and Management Areas of Calf Mortality in Large-Scale Dairy Herds
Source: Animals (Basel). 2025 Sep 24;15(19):2780. doi: 10.3390/ani15192780 (PMC12524161; doi:10.3390/ani15192780)
Supplement: Supplementary file 1 [file animals-15-02780-s001.zip › animals-3841179 Supplementary Table S3.pdf]

Supplementary Table S3. Descriptive statistics of continuous predictor variables and unconditional associations with herd annual calf mortality risk up to 21 days and 22-90 days of age in 118 Estonian dairy herds according to negative binomial regression analysis

| Block / Variable | Median | Quartiles | Incidence rate ratio for up to 21 day old calves' on-farm mortality risk | P-value[a] | Incidence rate ratio for 22-90 day old calves' on-farm mortality risk | P-value[a] |
|---|---|---|---|---|---|---|
| *Herd characteristics* | | | | | | |
| Herd average milk yield (×1000 kg) | 10429 | 9719; 11075 | 0.91 | 0.229 | 0.79 | <0.001 |
| *Colostrum management* | | | | | | |
| Duration (days) of colostrum or transition milk feeding to the calf | 3 | 2; 4 | 0.96 | 0.037 | 0.98 | 0.296 |
| Serum immunoglobulin G concentration (g/L) of up to seven day old calves | 14.6 | 10.8; 18.1 | 0.98 | 0.098 | 1.01 | 0.250 |
| *Calf feeding up to 21 days of age (post-colostrum)* | | | | | | |
| Maximum daily amount of milk or milk replacer fed | 7 | 6; 8 | 1.02 | 0.618 | 1.03 | 0.347 |
| Day of age since water is freely available | 3 | 1; 6 | 1.01 | 0.253 | 1.00 | 0.912 |
| *Calf housing during 5−21 days of age* | | | | | | |
| Frequency of bedding change for each calf (×3 times) | 1 | 0; 4 | 1.01 | 0.902 | 1.06 | 0.119 |
| *Calf feeding during 22−90 days of age* | | | | | | |
| Maximum daily amount of milk or milk replacer fed | 8 | 7; 9 | NA (NA) | NA | 1.08 | 0.030 |
| *Calf housing during 22−90 days of age* | | | | | | |

| | | | | | | |
|---|---|---|---|---|---|---|
| Frequency of bedding change for each calf (×10 times) | 7 | 2; 15 | NA (NA) | NA | 1.03 | 0.127 |

[a]Incidence rate ratio of the negative binomial regression model including herd number of cows as fixed effect.
