# Supplementary material for: Multiblock Analysis of Risk Factors and Management Areas of Calf Mortality in Large-Scale Dairy Herds
Source: Animals (Basel). 2025 Sep 24;15(19):2780. doi: 10.3390/ani15192780 (PMC12524161; doi:10.3390/ani15192780)
Supplement: Supplementary file 1 [file animals-15-02780-s001.zip › animals-3841179 Supplementary Table S4.pdf]

Supplementary Table S4. Variable importance indexes of the predictor variables in explaining composite outcome Y including herd annual calf mortality risk during the first 21 days and 22-90 days of age, and associations with the two fundamental variables of the Y, according to multiblock partial least squares analysis

Block / Variable name	Variable category	Composite Y VarImp index (95% CI)[a]	Association with individual Y variables[b]	
			On-farm mortality of up to 21 day old calves	On-farm mortality of 22-90 day old calves
Herd characteristics				
Herd predominant breed	Estonian Holstein Friesian breed ≥75%	0	0	0
	Estonian Red breed ≥75%	0.002 (-0.032; 0.004)	0.137 (-0.289; 0.512)	-0.022 (-0.390; 0.191)
	Mix of breeds	0.017 (-0.019; 0.035)	0.149 (-0.180; 0.619)	0.354 (0.117; 0.782)*
Perspective for changes in herd size	Increase	0	0	0
	Not to change	0.025 (-0.030; 0.050)	-0.277 (-0.621; 0.102)	-0.357 (-0.179; 0.091)
Milk yield	Herd average milk yield (×1000 kg)	0.036 (-0.018; 0.070)	-0.138 (-0.555; 0.200)	-0.539 (-1.016; -0.310)*
Time since building/reconstructing the calf barn	<10 years	0	0	0
	10−20 years	0.036 (-0.022; 0.069)	-0.507 (-0.873; -0.199)*	-0.179 (-0.472; 0.149)
	>20 years	0.019 (-0.022; 0.037)	0.403 (0.135; 0.787)*	0.029 (-0.310; 0.434)
Calving management				
Moving cow or heifer to the calving pen	Only for fetus ejection	0	0	0
	A few days before calving	0.001 (-0.008; 0.001)	-0.074 (-0.361; 0.126)	0.130 (-0.093; 0.355)
	More than a few days before calving	0.008 (-0.005; 0.015)	-0.171 (-0.410; 0.082)	-0.183 (-0.389; 0.018)
Time spent in the calving pen after calving	One hour or less	0	0	0
	Two to six hours	0.001 (-0.013; 0.002)	-0.124 (-0.388; 0.137)	0.043 (-0.201; 0.335)

	Seven to 48 hours	0.012 (-0.004; 0.023)	0.187 (-0.016; 0.396)	0.254 (0.065; 0.510)*
	Over 48 hours	0.001 (-0.009; 0.001)	-0.083 (-0.444; 0.115)	-0.018 (-0.308; 0.171)
Using technical tools to monitor calvings	No	0	0	0
	Yes	0.013 (-0.009; 0.026)	-0.215 (-0.428; 0.012)	-0.249 (-0.505; 0.021)
Cattle with dirty lower hind limb	<10%	0	0	0
	10−39%	0.002 (-0.011; 0.004)	0.062 (-0.213; 0.300)	0.108 (-0.098; 0.365)
	≥40%	0.012 (-0.008; 0.024)	0.227 (-0.043; 0.475)	0.223 (-0.004; 0.468)
Cattle with dirty udder	<10%	0	0	0
	≥10%	0.027 (0.004; 0.051)*	0.232 (-0.076; 0.442)	0.424 (0.165; 0.702)*
Cattle with dirty upper hind limb and flanks	<10%	0	0	0
	10−24%	0.024 (-0.013; 0.047)	-0.367 (-0.650; -0.040)*	-0.256 (-0.533; 0.078)
	25−49%	0.004 (-0.007; 0.007)	-0.103 (-0.334; 0.121)	-0.143 (-0.408; 0.077)
	≥50%	0.005 (-0.012; 0.010)	0.144 (-0.089; 0.372)	0.137 (-0.129; 0.415)
Colostrum management				
Duration of colostrum feeding	Duration (days) of colostrum or transition milk feeding to the calf	0.015 (-0.008; 0.029)	-0.324 (-0.598; -0.048)*	-0.191 (-0.446; 0.064)
Milking cows within two hours after calving	No	0	0	0
	Yes	0.000 (-0.017; 0.001)	0.059 (-0.260; 0.390)	0.019 (-0.306; 0.305)
	The calf suckles by itself	0.014 (-0.010; 0.027)	0.290 (0.093; 0.619)*	0.205 (-0.050; 0.567)
The amount of colostrum fed at first feeding	≤2 liters	0	0	0
	>2 liters	0.009 (-0.022; 0.018)	0.232 (-0.018; 0.584)	0.173 (-0.104; 0.544)
	Ad libitum	0.009 (-0.015; 0.017)	0.261 (0.080; 0.560)*	0.134 (-0.145; 0.475)

Measuring the quality of first colostrum	No (including farms where the calf suckles by itself)	0	0	0
	Yes	0.027 (-0.027; 0.053)	-0.298 (-0.635; -0.049)*	-0.402 (-0.743; -0.192)*
Using esophageal tube for first feeding if needed	No or sometimes	0	0	0
	Yes	0.013 (-0.028; 0.026)	-0.301 (-0.666; -0.061)*	-0.190 (-0.543; 0.095)
Calf housing during the first four days of life				
Individual box or group pen wall material	Laminated plywood	0	0	0
	Plastic	0.000 (-0.037; 0.000)	-0.047 (-0.455; 0.444)	0.010 (-0.375; 0.421)
	Metal	0.017 (-0.041; 0.034)	0.389 (0.048; 0.793)*	0.176 (-0.195; 0.595)
	Combined or other	0.005 (-0.021; 0.010)	-0.215 (-0.532; 0.181)	-0.086 (-0.414; 0.284)
Always washing calf box before placing a new calf	No	0	0	0
	Yes	0.057 (-0.044; 0.113)	-0.503 (-0.941; -0.250)*	-0.542 (-1.006; -0.286)*
Calf feeding up to 21 days of age (post-colostrum)				
Number of daily milk feedings	Two	0	0	0
	Three	0.015 (-0.009; 0.030)	-0.184 (-0.473; 0.011)	0.277 (0.066; 0.600)*
	Automatic milk feeder for part or whole period	0.001 (-0.010; 0.002)	0.023 (-0.352; 0.313)	-0.086 (-0.360; 0.078)
Equipment used for milk feeding	Individual nipple bucket	0	0	0
	Bucket	0.008 (-0.020; 0.016)	0.347 (0.043; 0.643)*	0.163 (-0.213; 0.489)
	Automatic milk feeder for part or whole period	0.015 (-0.009; 0.030)	-0.184 (-0.473; 0.011)	0.277 (0.066; 0.600)*
	Combined or other	0.004 (-0.012; 0.008)	0.136 (-0.156; 0.458)	-0.101 (-0.441; 0.178)

Feeding waste milk	No	0	0	0
	Yes	0.011 (-0.012; 0.020)	0.117 (-0.173; 0.436)	0.399 (0.183; 0.740)*
Silage freely available	No	0	0	0
	Yes	0.008 (-0.010; 0.015)	-0.254 (-0.507; -0.016)*	0.044 (-0.217; 0.346)
Calf housing during 5−21 days of age				
Prevailing keeping system and maximum group size	Individual pen	0	0	0
	Group pen, 2−12 animals per pen	0.002 (-0.012; 0.005)	0.155 (-0.091; 0.374)	0.019 (-0.237; 0.243)
	Group pen, ≥13 animals per pen	0.005 (-0.010; 0.009)	0.022 (-0.283; 0.242)	0.231 (0.024; 0.493)*
Calf pen floor material	Solid or slatted wood	0	0	0
	Concrete	0.003 (-0.008; 0.007)	-0.082 (-0.378; 0.176)	0.151 (-0.097; 0.385)
	Mat or mattress	0.003 (-0.006; 0.007)	0.141 (-0.096; 0.418)	-0.097 (-0.399; 0.139)
	Combined or other	0.007 (-0.005; 0.013)	-0.059 (-0.244; 0.116)	0.246 (0.031; 0.599)*
Always washing calf pen before placing a new calf	No or sometimes	0	0	0
	Always	0.020 (-0.004; 0.037)	0.229 (-0.013; 0.498)	0.452 (0.238; 0.745)*
	No calf movements during this period	0.001 (-0.009; 0.003)	-0.108 (-0.338; 0.093)	-0.077 (-0.349; 0.171)
Bedding change	Number of bedding changes during 5−21 days (×3 times)	0.004 (-0.014; 0.008)	0.071 (-0.201; 0.289)	0.206 (-0.073; 0.514)
Possibility to defecate into another pen	No	0	0	0
	Yes	0.004 (-0.009; 0.007)	0.110 (-0.150; 0.349)	0.191 (0.015; 0.446)*
Forced air ventilation in calves' room	No	0	0	0
	Yes	0.011 (-0.006; 0.020)	-0.186 (-0.486; 0.053)	-0.326 (-0.586; -0.136)*

Access to an outdoor area	No	0	0	0
	Yes	0.014 (0.004; 0.026)*	-0.236 (-0.538; -0.041)*	0.249 (0.017; 0.569)*
Calf feeding during 22−90 days of age				
Feeding system	Individual feeding, two or three times per day	0	0	0
	Automatic milk feeder	0.014 (-0.025; 0.027)	0.281 (0.033; 0.755)*	-0.257 (-0.656; 0.025)
	Combined (individual feeding + automatic feeder)	0.004 (-0.107; 0.008)	-0.003 (-0.636; 0.507)	0.228 (-0.270; 0.882)
Maximum daily feed amount during this period	Maximum amount of milk or milk replacer	0.022 (-0.052; 0.044)	0.153 (-0.246; 0.479)	0.525 (0.203; 0.979)*
Silage freely available	No	0	0	0
	Yes	0.019 (-0.056; 0.038)	-0.371 (-0.793; 0.025)	-0.356 (-0.759; -0.016)*
Calf housing during 22−90 days of age				
Number of animals in one group pen	No group pens	0	0	0
	≤15 animals per pen	0.011 (-0.006; 0.022)	-0.114 (-0.436; 0.120)	0.324 (0.087; 0.637)*
	≥16 animals per pen	0.001 (-0.025; 0.001)	-0.047 (-0.437; 0.291)	-0.100 (-0.497; 0.196)
Principle of adding calves to group pens	As a group	0	0	0
	Continuously	0.003 (-0.011; 0.005)	-0.021 (-0.355; 0.203)	-0.206 (-0.525; -0.019)*
	No group pens	0.001 (-0.025; 0.001)	-0.047 (-0.437; 0.291)	-0.100 (-0.497; 0.196)
Maximum age difference of calves in the same pen	2−9 days	0	0	0
	10−19 days	0.014 (-0.004; 0.027)	-0.321 (-0.667; -0.062)*	0.156 (-0.117; 0.464)

	20−39 days	0.002 (-0.013; 0.004)	0.123 (-0.081; 0.407)	0.197 (0.020; 0.500)*
	≥40 days	0.007 (-0.009; 0.014)	-0.027 (-0.272; 0.193)	-0.327 (-0.617; -0.130)*
	No group pens	0.001 (-0.025; 0.001)	-0.047 (-0.437; 0.291)	-0.100 (-0.497; 0.196)
Forced air ventilation in calves' room	No	0	0	0
	Yes	0.002 (-0.014; 0.005)	-0.181 (-0.509; 0.057)	-0.175 (-0.473; 0.017)
Routine stress-inducing activities				
Disbudding calves	Only heifer calves	0	0	0
	Heifer and bull calves	0.052 (-0.037; 0.100)	0.619 (0.233; 1.057)*	0.209 (-0.249; 0.648)
Age of calves at disbudding	≤ 20 days	0	0	0
	21−29 days	0.046 (-0.058; 0.089)	0.577 (0.260; 1.072)*	0.130 (-0.339; 0.630)
	≥30 days	0.054 (-0.028; 0.106)	-0.583 (-1.162; -0.293)*	0.062 (-0.341; 0.497)
General disease prevention measures				
Employing wet disinfection in calving pen	No	0	0	0
	Yes	0.010 (-0.033; 0.019)	-0.292 (-0.592; 0.047)	-0.177 (-0.569; 0.253)
Using calving pens for sick or soon to be culled cows	No	0	0	0
	Yes	0.010 (-0.021; 0.019)	0.028 (-0.333; 0.339)	0.382 (0.040; 0.738)*
Drying calves after birth	No	0	0	0
	Yes, seasonally	0.004 (-0.015; 0.008)	0.037 (-0.318; 0.303)	0.230 (-0.078; 0.534)
	Yes, always	0.009 (-0.018; 0.018)	0.145 (-0.193; 0.470)	0.313 (0.060; 0.611)*
	No	0	0	0

Using wet disinfection in newborn calves' boxes	Yes	0.004 (-0.019; 0.008)	-0.057 (-0.525; 0.198)	-0.229 (-0.726; -0.023)*
Preventively using injectable antibiotics in calves	No	0	0	0
	Yes, during some period	0.015 (-0.013; 0.030)	-0.256 (-0.502; 0.059)	-0.350 (-0.636; -0.028)*
	Yes, continuously	0.004 (-0.013; 0.007)	0.218 (-0.075; 0.499)	0.039 (-0.294; 0.273)
Pathogen-specific disease prevention measures				
Routine usage of oral anti-cryptosporidiosis drugs	No	0	0	0
	Yes	0.013 (-0.033; 0.025)	0.321 (-0.130; 0.630)	0.219 (-0.293; 0.522)
Vaccinating calves against bovine herpesvirus 1	No	0	0	0
	Yes	0.013 (-0.027; 0.026)	-0.128 (-0.419; 0.144)	-0.376 (-0.725; -0.184)*
Vaccinating calves against bovine viral diarrhea virus	No	0	0	0
	Yes	0.013 (-0.027; 0.026)	-0.128 (-0.419; 0.144)	-0.376 (-0.725; -0.184)*
Vaccinating cows/heifers against bovine herpesvirus 1	No	0	0	0
	Yes	0.013 (-0.010; 0.025)	-0.270 (-0.535; -0.084)*	-0.281 (-0.684; -0.043)*
Vaccinating cows/heifers against parainfluenza virus 3	No	0	0	0
	Yes	0.011 (-0.037; 0.021)	0.112 (-0.106; 0.326)	-0.319 (-0.788; -0.111)*
Vaccinating cows/heifers against *Mannheimia haemolytica*	No	0	0	0
	Yes	0.011 (-0.037; 0.021)	0.112 (-0.106; 0.326)	-0.319 (-0.788; -0.111)*
Disease status				
	Negative	0	0	0

Herd bovine respiratory syncytial virus status	BTM positive, heifers negative	0.005 (-0.041; 0.009)	-0.307 (-0.680; 0.050)	-0.181 (-0.584; 0.310)
	BTM and heifers positive	0.002 (-0.026; 0.004)	-0.207 (-0.623; 0.109)	-0.066 (-0.477; 0.284)
	Vaccinating herds	0.001 (-0.045; 0.003)	-0.176 (-0.516; 0.390)	-0.050 (-0.353; 0.382)
Calf faecal samples positive for bovine rotavirus	No	0	0	0
	Yes	0.006 (-0.037; 0.012)	-0.001 (-0.388; 0.444)	-0.400 (-0.860; -0.038)*
External biosecurity				
Grazing cows and/or heifers	No	0	0	0
	Only youngstock	0.011 (-0.008; 0.021)	-0.223 (-0.486; 0.030)	-0.278 (-0.541; -0.043)*
	Only cows (including only dry cows)	0.000 (-0.011; 0.001)	-0.047 (-0.361; 0.216)	-0.056 (-0.368; 0.160)
	Dry cows and youngstock	0.016 (-0.008; 0.032)	0.194 (-0.022; 0.500)	0.401 (0.221; 0.861)*
Participation in animal shows during the last 3 years	No	0	0	0
	Yes	0.003 (-0.007; 0.006)	0.125 (-0.096; 0.377)	-0.164 (-0.431; 0.031)
Farm workers change clothes on the farm	No or sometimes	0	0	0
	Yes	0.006 (-0.012; 0.011)	-0.124 (-0.395; 0.185)	-0.232 (-0.514; 0.016)
Visitors wear protective clothing and footwear	No or sometimes	0	0	0
	Yes	0.009 (-0.010; 0.016)	0.005 (-0.250; 0.352)	-0.336 (-0.661; -0.066)*
Disinfection mats/baths at people's entry points	No	0	0	0
	Yes	0.002 (-0.007; 0.004)	-0.027 (-0.295; 0.175)	-0.162 (-0.432; 0.022)
Distance between farm and carcass loading place	≤ 5 m	0	0	0
	6−19 m	0.004 (-0.010; 0.008)	0.216 (-0.020; 0.495)	0.048 (-0.212; 0.282)
	20−49 m	0.010 (-0.013; 0.020)	-0.334 (-0.597; -0.020)*	-0.098 (-0.379; 0.304)

≥50 m	0.003 (-0.011; 0.005)	-0.118 (-0.469; 0.151)	-0.132 (-0.401; 0.099)

[a] Variable importance index estimates and ninety-five percent confidence intervals. Asterisk (*) indicates significant contribution (95% confidence interval not containing zero) in explaining the composite mortality estimate, composed of herd annual calf mortality during the first 21 days and mortality during 22-90 days of age.

[b] Association between predictor variables with each outcome variable in the Y block, based on the regression coefficient β and ninety-five percent confidence intervals. Asterisk (*) indicates significant contribution (95% confidence interval not containing zero).
